# Targeted therapeutic hypothermia protects against noise induced hearing loss

**DOI:** 10.3389/fnins.2023.1296458

**Published:** 2024-01-16

**Authors:** Samantha Rincon Sabatino, Andrea Rivero, Rachele Sangaletti, W. Dalton Dietrich, Michael E. Hoffer, Curtis S. King, Suhrud M. Rajguru

**Affiliations:** ^1^Department of Biomedical Engineering, University of Miami, Coral Gables, FL, United States; ^2^Department of Otolaryngology, University of Miami, Coral Gables, FL, United States; ^3^The Miami Project to Cure Paralysis, University of Miami, Coral Gables, FL, United States; ^4^RestorEar Devices LLC, Bozeman, MT, United States

**Keywords:** noise-induced hearing loss, hidden hearing loss, therapeutic hypothermia, neuroprotection, synaptopathy, hair cells

## Abstract

**Introduction:**

Exposure to occupational or recreational loud noise activates multiple biological regulatory circuits and damages the cochlea, causing permanent changes in hearing sensitivity. Currently, no effective clinical therapy is available for the treatment or mitigation of noise-induced hearing loss (NIHL). Here, we describe an application of localized and non-invasive therapeutic hypothermia and targeted temperature management of the inner ear to prevent NIHL.

**Methods:**

We developed a custom-designed cooling neck collar to reduce the temperature of the inner ear by 3–4°C post-injury to deliver mild therapeutic hypothermia.

**Results:**

This localized and non-invasive therapeutic hypothermia successfully mitigated NIHL in rats. Our results show that mild hypothermia can be applied quickly and safely to the inner ear following noise exposure. We show that localized hypothermia after NIHL preserves residual hearing and rescues noise-induced synaptopathy over a period of months.

**Discussion:**

This study establishes a minimally-invasive therapeutic paradigm with a high potential for rapid translation to the clinic for long-term preservation of hearing health.

## 1 Introduction

Hearing loss (HL) is a global public health concern, afflicting an estimated 5% of the world population with a projected prevalence of 10% by 2050 (World Health Organization, 2021). One of the most prevalent causes of permanent sensorineural damage is avoidable noise overexposure, which impairs hearing and increases the risks of tinnitus (Bramhall et al., 2018) and vestibular dysfunction (Wang and Young, 2007). Despite the preventability of NIHL, recommended occupational and recreational noise exposure levels are frequently breached, resulting in a high risk for permanent hearing damage for children to adults (Tak et al., 2009; Mahboubi et al., 2013; World Health Organization, 2016; Green et al., 2021). Preclinical studies in mammalian models have proposed that moderate exposures resulting in only temporary loss of hearing function may confer veiled permanent damage to synaptic connections (Kujawa and Liberman, 2009; Lin et al., 2011; Jensen et al., 2015), which in turn may accelerate age-related changes in hearing (Fernandez et al., 2015).

Despite the global burden of NIHL, current prevention methods are limited to precautions such as withdrawal from noisy environments or the use of hearing protection devices (HPD), which are routinely ignored (Tak et al., 2009; Yankaskas, 2013). HPDs can attenuate situational awareness, which may reduce compliance to using them as standard personal protective equipment. (Snapp et al., 2023). Various promising pharmacologic agents have also been identified for NIHL intervention, including antioxidants (Bielefeld et al., 2007; Campbell et al., 2011; Fetoni et al., 2013), glutamate antagonists (Hu et al., 2020), cell death regulators (Wilson et al., 2014), and anticonvulsive drugs (Bao et al., 2013). However, successful clinical translation has been hampered by the complexity of implicated cellular and molecular pathways. Currently none of these agents have FDA-approval for hearing loss prevention or rescue (Wang and Puel, 2018; Le Prell et al., 2019). These disappointing outcomes of preclinically promising targets are due in part to challenges selecting the optimal agents, their dosages and delivering the compounds to the cochlea. The duration and timing of application post-injury remain a challenge as the compounds administered after noise exposure may not act fast enough during the critical window for protection. The feasibility of timely delivery of intracochlear or systemic therapeutics may also limit translation to the general population. Alternatively, several stem cell-based therapies and AAV-vector or CRISPR-Cas9-based gene therapies are also being investigated. However, they are still in preclinical development (Schilder et al., 2019; Nourbakhsh et al., 2021; Stojkovic et al., 2021; Zine et al., 2021). To address the rising global challenge of hearing loss, there is a need to consider therapeutic strategies with demonstrated success in areas of neurotrauma.

Mild therapeutic hypothermia (MTH) for neuroprotection has been extensively studied in traumatic brain injury, stroke, and spinal cord injuries. MTH modulates inflammatory and apoptotic pathways (Schmitt et al., 2014), reduces free radical production (Lee et al., 2009), and attenuates infiltration of circulating monocytes (Lotocki et al., 2009). Several studies have also documented the physiological effects of dysregulated cochlear temperature on auditory responses (Brown et al., 1983; Ohlemiller and Siegel, 1992), promoting the hypothesis that hypothermic intervention after cochlear injury may provide a protective benefit. In a study conducted in mice, systemic cooling and warming during noise exposure elicited differential responses in acoustic injury and recovery (Henry, 2003). While hyperthermia (40°C) augmented hearing threshold shifts, hypothermia (30°C) allowed retention of residual hearing capability after noise. Similar benefits have been observed for cochlear ischemic injury (Hyodo et al., 2001). Although these studies showed benefits of MTH, systemic application can result in unintended complications and is simply not feasible as a therapeutic application for a global population. Recent studies in cochlear implant trauma (Tamames et al., 2016; Bader et al., 2023; Schmutzhard et al., 2023) and cisplatin injury (Spankovich et al., 2016; Stanford et al., 2020) models provide evidence for localized MTH providing cochlear neuroprotection. Furthermore, several approaches have been proposed to achieve therapeutic levels of hypothermia (Spankovich et al., 2016; Tamames et al., 2016, 2018; Bader et al., 2020; Stanford et al., 2020; Arteaga et al., 2023). However, feasibility and application of localized MTH to mitigate effects of noise exposure have yet to be studied.

In this study, we demonstrate a non-invasive and controlled localized induction of MTH within the inner ear in a preclinical model. We further characterize efficacy of MTH as a potential approach for preventing NIHL in a preclinical model. Hearing preservation was assessed by comparison of auditory brainstem response (ABR) thresholds within an acute recovery phase of 28 days-post noise as well as in long-term aging studies up to 12 months post-noise. We further demonstrate the beneficial effects of local hypothermia treatment for maintaining normal cochlear structure via immunohistological evaluation of hair cell and synapse integrity and alterations in ABR morphology.

## 2 Materials and methods

### 2.1 Experimental design

All procedures were approved by the University of Miami Animal Care and Use Committee. Brown Norway rats aged between 15 and 20 weeks (Charles River Laboratories) were initially screened for normal baseline hearing thresholds (<30 dB for 4–32 kHz, <50 dB for click and 2 kHz) prior to group block randomization.

Two studies were conducted in parallel:

- One to establish the early efficacy of mild therapeutic hypothermia post-noise exposure and
- Second to establish the efficacy of one-time hypothermic application in a long-term study.

**Figure 1:**
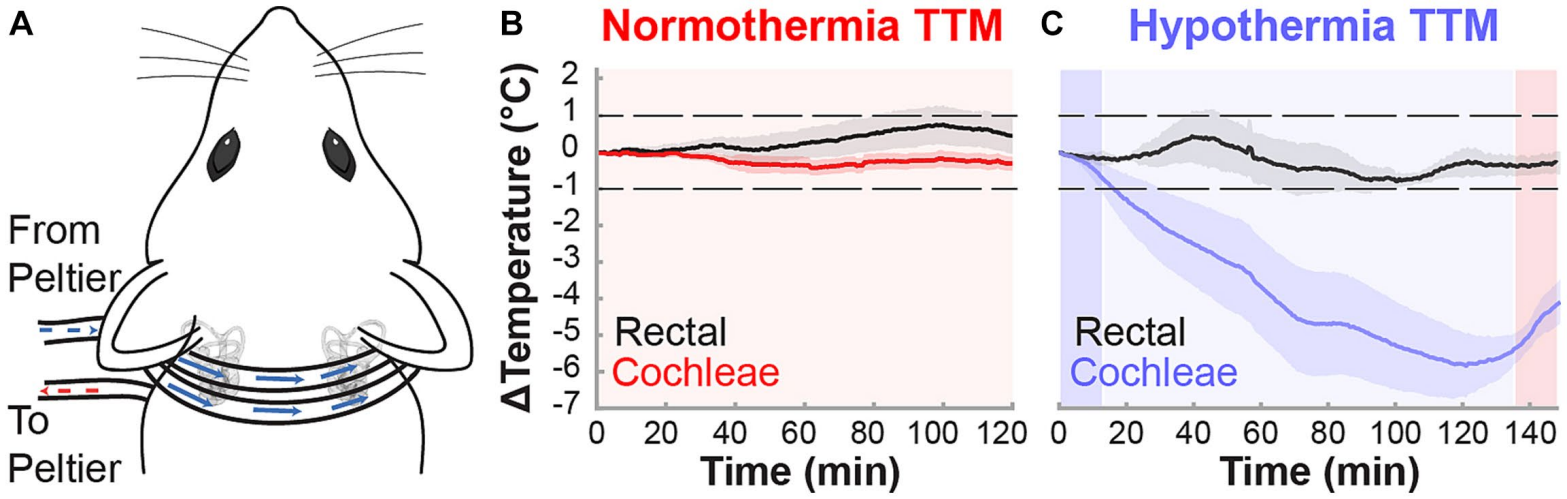
Non-invasive targeted cochlear temperature management. **(A)** The cooling neck collar placement was maintained above rat bullae for the duration of the TTM protocols to deliver mild therapeutic hypothermia. Validation of TTM target temperatures was performed in acute experiments (*n* = 3, *k* = 6 cochleae) measuring intracochlear and rectal temperatures (black trace) during the normothermia **(B)** and hypothermia **(C)** protocols. Temperature data are presented as mean ± SE.

For the first study establishing the early effects of NIHL and the potential benefit of hypothermic neuroprotection, female Brown Norway rats (*n* = 18) were randomly placed into three groups: *Noise only (noise exposure but no targeted temperature management)*, *Noise + Normothermia (noise followed by targeted temperature management (TTM) at ~ 37°C)*, and *Noise + Hypothermia (noise followed by TTM at ~ 33°C)*. All animals were subjected to 2 h of continuous noise under isoflurane anesthesia in euthermic (~37°C) conditions. The noise only group recovered in euthermic awake conditions following noise exposure. Conversely, the animals receiving normothermia or hypothermia treatments were kept anesthetized using intramuscular injection of ketamine (44 mg/kg) and xylazine (5 mg/kg) for a 2-h recovery period with either localized normothermia (~37°C) or hypothermia (~33°C) TTM. The TTM was achieved with a custom circulating coolant system and a neck collar (discussed below, Figure 1). Animals used for functional hearing assessment were monitored for hearing and behavioral changes up to the study endpoint at 28 days post-noise. A separate set of female animals was used for detailed functional and histological assessment of synapses (*n* = 16, 24-h post-noise). Animals including age-matched unexposed controls (*n* = 4) were anesthetized with ketamine/xylazine (44/5 mg/kg) for transcardial fixation prior to cochlear extraction for immunohistology.

For the second study evaluating the long-term efficacy and safety of hypothermic intervention, age-matched male and female Brown Norway rats (*n* = 30) were randomly placed into three groups: *Hypothermia Control (no noise, 33°C)*, *Noise + Normothermia (37°C)*, and *Noise + Hypothermia (33°C)*. The *Hypothermia Control* group was used to verify the safety of cooling the inner ear by delivering MTH using the same approach but without any form of trauma. This group further allowed study of potential adverse effects of MTH on hearing function. Noise and TTM procedures remained unchanged from the acute phase study. Changes in animal’s hearing functions were measured up to 12 months post-noise. At the study endpoint, inner ear extraction was performed following transcardial fixation. This study utilized age-matched male and female animals to understand the influence of biological sex on hypothermic neuroprotection.

### 2.2 Hearing tests

ABR tests were performed to quantify changes in hearing threshold in anesthetized rats following the noise exposure. ABR measurements were also carried out prior to noise exposure to establish a baseline. Early time points of 1, 3-, 7-, 14-, and 28-days post-noise (DPN) were used to analyze ABR threshold progression within the acute recovery phase. In additional animals, time points of 2-, 3-, 6-, 9-, and 12-months post-noise (MPN) were assessed for long-term recovery and safety of the hypothermic intervention.

ABR assessment was performed in rats anesthetized with intramuscular injection of ketamine (44 mg/kg) and xylazine (5 mg/kg). Animals were maintained at ~37°C with heating pads throughout the testing period. The rats were placed within a custom-designed soundproof chamber (Med Associates Inc., Fairfax, VA). Acoustic stimuli were delivered through earphones placed in the external ear canal. ABRs were recorded using subcutaneous needle electrodes attached to a pre-amplifier and a data acquisition system (Intelligent Hearing Systems, FL IHS SmartEP software, ER2 and ER3 High-Frequency Transducers). Subcutaneous needle electrodes were placed next to each bulla, with a reference electrode placed at the vertex and a ground electrode in a hind leg muscle. The system’s sampling rate is 128 kHz, sufficient to generate stimuli up to 32 kHz. Each transducer was calibrated for intensities at each frequency and accounted for the species’ ear volumes and canal size. Finally, high-pass filters were used to reduce any harmonic distortion.

Pure–tone hearing thresholds were tested at 2, 4, 8, 16, 24, and 32 kHz. The broadband frequency threshold was also determined with a click stimulus of 1,000 μs. ABR testing was initiated with 1,024 sweeps at 80 dB SPL (Sound Pressure Level) and was reduced by 10 dB steps until a threshold was observed. ABR thresholds were determined as the minimum stimulating SPL to elicit a recognizable Wave I peak. Hearing threshold shifts were then determined by subtracting Baseline thresholds from post-noise or post-procedure thresholds. For ABR thresholds that exceeded the 90 dB SPL predetermined testing safety limit, the threshold shift was calculated using 90 dB as the estimated threshold. ABR waveform analysis was performed at select frequencies and intensities to determine post-noise Wave I and IV node-to-peak amplitudes and latency changes. Obtained ABR thresholds, Wave I, and Wave IV amplitudes and latencies were verified by blinded investigators.

### 2.3 Noise exposure

Rats were placed in a soundproof chamber (IHS) and exposed to 2 h of continuous noise. The acoustic noise was generated in MATLAB (R2017a) and processed in Audacity (2.1.2) to form a 2-h long, monaural continuous 4–8 kHz narrowband signal. The acoustic signal was emitted from an overhead speaker (Pyle PDBT45) driven by an amplifier (Pyle PPA450). Sound levels were calibrated throughout the soundproof chamber with SPL meters (Extech 407732, UT353 BT) to deliver noise at 105 dB and were checked every 15 min during noise exposure. Noise was delivered one animal at a time, with the animal under isoflurane anesthesia (4% for induction and 1.5% for maintenance with 1% Oxygen).

### 2.4 Non-invasive cochlear targeted temperature management

The custom cooling device utilized has been detailed for localized cooling during cochlear implant surgeries (Tamames et al., 2016). The device modifications here included a surface cooling attachment (neck collar) to induce mild hypothermia bilaterally in the rat cochleae non-invasively to reach desired temperatures of 31–33°C. The surface cooling attachment consisted of a 0.11” ID × 0.15” OD silicon collar used to circulate fluorocarbon, an inert refrigerant, controlled by a thermoelectric Peltier system (Figure 1A). Hypothermia and normothermia TTM protocols were performed on anesthetized animals for a minimum 2-h duration. The normothermia TTM protocol consisted of a controlled neck collar temperature maintenance (fluorocarbon: 33°C, inner ear or cochlear temperature maintained ~37°C) for 2 h (Figure 1B). The hypothermia TTM protocol (Figure 1C) consisted of an initial 12-min induction period (fluorocarbon temperature decrease: 33°C to 5°C), followed by a 2-h continuous cooling period (fluorocarbon stable temperature: 5°C, inner ear or cochlear temperature maintained ~33°C), and finalized with a 12-min rewarming period (fluorocarbon temperature increase: 5°C to 33°C).

### 2.5 Acute intracochlear measurements

In acute experiments, intracochlear temperatures were measured using microthermistors (Omega, 5SC-TT-T-40-36) surgically implanted through the round window. MTH was delivered with the custom device using the protocol outlined above, Temperature changes from baseline were averaged between left and right cochleae of individual rats. Rectal temperature was measured and body maintained at 37°C for the protocol duration.

### 2.6 Immunohistochemistry

Transcardial fixation was performed with 1% phosphate-buffered saline (PBS) initial perfusion followed by 4% paraformaldehyde (PFA). Cochleae were then harvested and intracochlearly perfused with 4% PFA and fixed overnight. Cochleae were then decalcified by 48-h incubation in 10% ethylenediaminetetraacetic acid (EDTA, in PBS). Following decalcification, cochlear samples were washed (PBS, 3x, 10 min) and stored in PBS for subsequent organ of Corti dissections.

**Figure 2:**
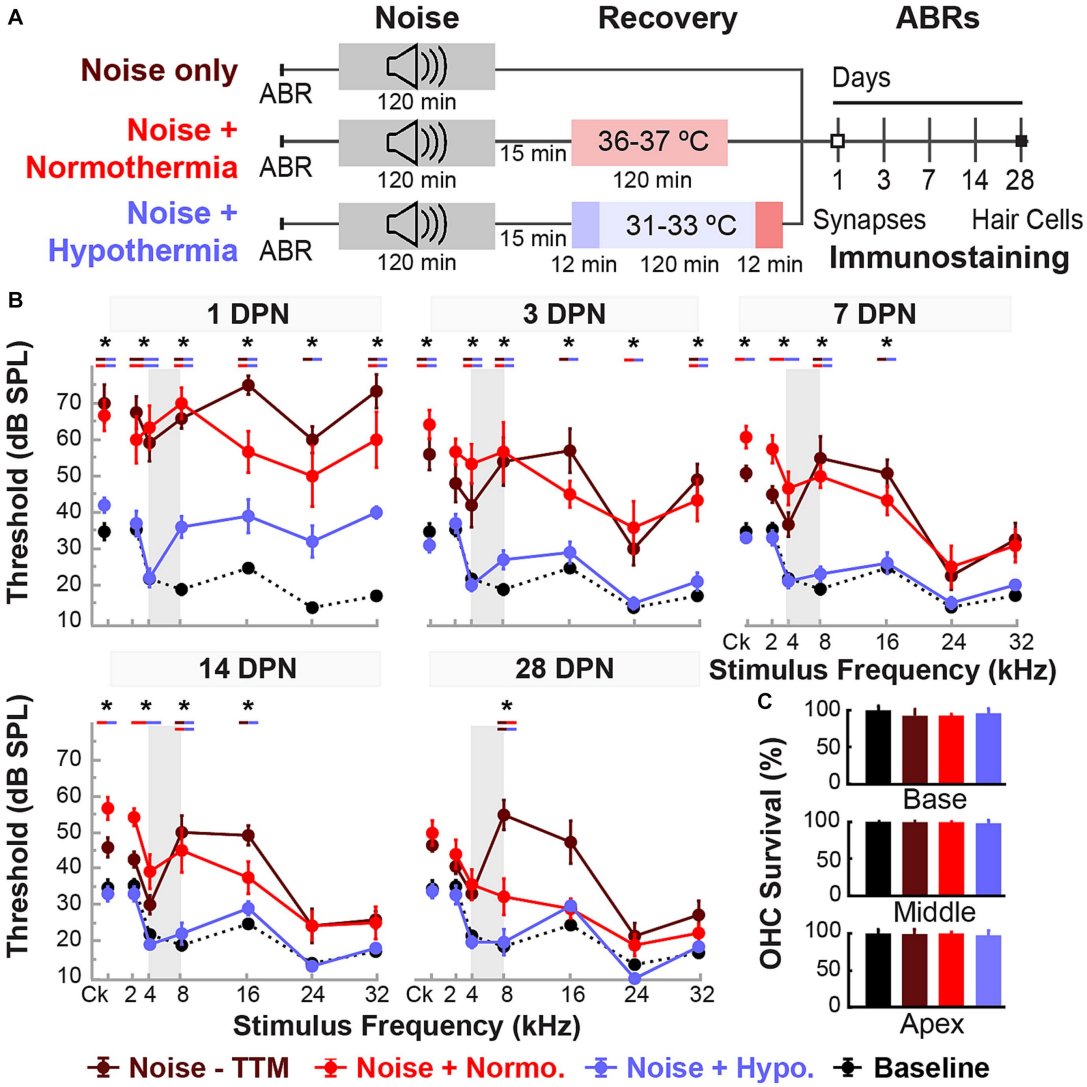
Post-noise hypothermia attenuates early ABR hearing threshold shifts. **(A)** Experimental protocol for animals used to study of recovery of noise-induced induced injury with TTM. Animals were exposed to 2 h of continuous noise at 105 dB under isoflurane anesthesia before initiating TTM protocols of normothermia and hypothermia. For animals receiving TTM, temperature modulation commenced roughly 15 min post-noise. **(B)** ABR thresholds were obtained in age-matched female rats at 1, 3, 7, 14 and 28 DPN and compared to combined baseline thresholds (dashed curve). ABRs were obtained for click (Ck) and 2, 4, 8, 16, 24, and 32 kHz pure-tone stimuli. The frequency spectrum of the experimental noise is illustrated by gray vertical bars at 4–8 kHz. Results for post-noise ABR thresholds are shown for animals that recovered from noise exposure in *noise only* (dark red), *normothermia TTM* (red), and *hypothermia TTM* (light blue) conditions. Data presented as mean ± SE of ABR thresholds with between-group comparisons illustrated with colored bars at each stimulus frequency (*p < 0.05, **p < 0.01). **(C)** To assess hair cell integrity after noise exposure, FITC-Phalloidin labeled hair cell survival was quantified at 28 DPN for apical, middle, and basal cochlear regions in unexposed control (black), noise only (dark red), noise+normothermia (red), and noise+hypothermia animals (blue). Hair cell survival data for respective groups are shown as percent survival (mean ± SE).

Samples used for hair cell counts at 28 DPN (*n* = 18, Recovery Phase detailed in Figure 2A) were first permeabilized with 0.3% Triton X-100 (in PBS, 1 h) before washing and subsequent incubation in 5% normal horse serum (NHS, in PBS, 1 h) to prevent non-specific binding. After washing, samples were incubated with FITC-labeled Phalloidin (1:100, in PBS, 30 min) and DAPI (1:1000, in PBS, 10 min) before cochlear whole mount preparation.

Cochlear specimens used for hair cell and ribbon synapse labeling at 1 DPN (*n* = 16, 4/group, Figure 2A) were first permeabilized by freezing and thawing samples in 30% sucrose. After washes (PBS, 3x, 10 min), samples were incubated with 5% NHS (in PBS, 1 h). For primary antibody incubation, samples were incubated overnight at 37°C with rabbit anti-MyosinVIIa (1:200, ab3481), mouse (IgG1) anti-CtBP2 (1:200; BD Transduction Laboratories #612044), and mouse (IgG2a) anti-GluA2 (1:2000, Millipore #MAB397) in 1% NHS (in PBS, with 0.1% Triton X-100) to label hair cell bodies and pre-synaptic densities. Following washing steps, the samples were incubated at 37°C with corresponding secondary antibodies chicken anti-rabbit (IgG)-647 (1:200, A-21443), goat anti-mouse (IgG1)- 568 (1:1000, A-21124), and goat anti-mouse (IgG2a)-488 (1:1000, A-21131) in 1% NHS (in PBS, with 0.1% Triton X-100, 1 h, washed and repeated). Immunolabeled samples were then mounted with Fluoromount Aqueous Mounting Medium (Sigma F4680) and covered with a coverslip to be imaged.

For assessment of delayed degeneration of SGN at 12 months following acute acoustic over-exposure (*n* = 30, 5/sex/group, Aging Phase detailed in Figure 2A), whole inner ear samples with an attached vestibular labyrinth and otic capsule were first oriented to provide clear mid-modiolar sections across the basal and middle turns and subsequently placed in standard plastic cassettes for serial dehydration steps from 70 to 100% ethanol (190, 200 Proof Greenfields Global) at 45-min wash cycles. The samples were then embedded in 60°C paraffin (Paraplast X-TRA) and thereafter stored at room-temperature until the solid-embedded samples were serially cut under heated water bath to obtain sections at a thickness of 10 μm. Sectioned samples were placed on glass slides and every fifth sample was prepped for hematoxylin and eosin (H&E) staining. Staining preparation commenced with deparaffinization consisting of initial heating in an oven at 60°C for 30 min, followed by xylene incubation (3 min, 3x) before serial rehydration steps from 100 to 95% ethanol in 30 s wash cycles. Afterwards, H&E staining was performed with a progressive method, including incubation in Gill’s Hematoxylin II (Polyscience Inc., #S210, 1 min, 2x), dH_2_O rinse (3 min, 2x), and Eosin Phloxine (Polyscience Inc., #S176, 3 min). Samples were again dehydrated in 1-min repeated wash cycles from 95 to 100% ethanol before final xylene clearing (2 min, 2x). Sectioned samples were then evaluated under light microscopy and selected slides containing clear mid-modiolar cochlear morphology were evaluated for histological analysis.

### 2.7 Hair cell, ribbon synapse, and spiral ganglion neuron quantification

Mounted fluorescence samples for observation of hair cells and ribbon synapses were imaged with a Zeiss LSM 700 confocal microscope. Brightfield images of cochlear whole mount samples were imaged for cochlear frequency mapping using a Measure Line ImageJ plugin developed by Massey Eye-Ear Institute’s Eaton-Peabody Laboratory.^1^ For IHC and OHC counts observed at 28 DPN, confocal z-stacks were obtained for the representative cochlear apex, middle, and basal regions at 40X magnification using an oil-immersion objective. Hair cell counts were then performed by blinded investigators using ImageJ software and expressed as a percentage of HC survival assessed from DAPI-labeled nuclei and FITC-Phalloidin labeled hair cell cuticular plates. The percentage of outer hair cell (OHC) survival was assessed along 200 μm of sensory epithelium in representative basal, middle, and apical regions.

For cochlear ribbon synapses observed at 1 DPN, a detailed view of the IHC synaptic region was obtained using a 63X oil-immersion objective. Z-stacks for ribbon counts were performed at 0.2 μm intervals at tonotopic frequency locations of 8 and 16 kHz, as determined using the cochlear frequency mapping ImageJ plugin (see text footnote 1). Stacks were obtained from the point of visible MYosinVIIa-labeled inner hair cell body to final CtBP2-labeled ribbon disappearance. Confocal z-stacks obtained for ribbon counts were first processed with ImageJ (Schneider et al., 2012) to crop the image area to contain 10 IHCs identified by Myosin-VIIa and establish the threshold for CtBP2-labeling of IHC nuclei, highlighting the CtBP2-labeled presynaptic ribbons. Next, paired ribbon synapse counts were assessed by visual inspection of postsynaptic GLUR2-labeled puncta and presynaptic CtBP2-labeled puncta performed by blinded assessment through SyGlass (v1.7.2) 3D visualization software. The Surfaces Tool in SyGlass was used to first create volumetric meshes of the GLUR2- and CtBP2-labeled puncta and determine volumetric location and size. Quantification of paired and orphan GluR2 and CtBP2 puncta was then performed using the Counting Tool in SyGlass and exported for statistical analysis.

For the 12 MPN cochlear sections used for SGN density estimations, the 10 μm paraffin-sectioned samples were imaged using an Olympus BX50 light microscope equipped with Stereoinvestigator. Samples were imaged using 20X objectives and stitched *post hoc*. SGN densities were then computed for mid-modiolar sections of the basal and middle turns using ImageJ software to identify SGN with clear nuclei and cytoplasm within Rosenthal’s canal. Measurements for SGNs and Rosenthal’s Canal area were determined through blinded investigator evaluation.

### 2.8 Statistical analysis

Data from blinded ABR evaluation of thresholds and wave demarcation were imported into SAS JMP (JMP PRO 15) for statistical analysis. Obtained ABR data was binaurally averaged to limit the pseudoreplication of the biological variable. Early group-specific threshold outliers were investigated at 1 DPN, removing all further subject data from statistical models. ABR thresholds and amplitudes were compared between experimental groups using linear mixed-effects regression models to account for missing longitudinal data with repeated measures. Unless otherwise specified, fixed model effects included treatment, stimulation frequency, and time along with their two- and three-way interactions. Random subject effects were included, utilizing unbounded variance only when providing significant model benefit. Model residuals were used to validate data normality assumptions with a visual inspection of quantile-quantile plots and homogeneity of variance with Levene’s test. Bonferroni corrections were used for planned *post hoc* between-group comparisons at specified study endpoints. To minimize physiological variability, Wave I and IV amplitudes were normalized to baseline amplitude for same respective ears and stimuli at the highest experimental stimulus level (80 dB) for 8 and 16 kHz pure-tone stimuli. Post-procedure normalized wave percentages at a suprathreshold level of 80 dB were compared between experimental groups at distinct time points using mixed-effects models, including random subject effects and fixed frequency, time, and treatment effects. Immunohistological measures of hair cell survival, synapse densities, and spiral ganglion densities were assessed using two-way ANOVA (treatment x cochlear location) and *post hoc* Tukey’s test. In addition, angular transformations were performed on all data normalized as percent survival within a survival range from 80 to 100%. For all illustrated comparisons, data are presented as group means ± SEM with significant differences presented as (*) for *p* < 0.05 and (**) for *p* < 0.01, non-significant differences are marked as n.s.

## 3 Results

### 3.1 Evaluation of non-invasive TTM in the inner ear

Localized and controlled TTM to the inner ear was induced in anesthetized (ketamine, xylazine) male and female Brown Norway rats through a neck collar attachment that was applied to a previously designed system (Figure 1A; Tamames et al., 2016). Target temperatures were validated in acute experiments (*n* = 3), where induced temperature changes were measured rectally to examine systemic effects and intracochlearly using micro-thermocouples implanted via the round window and fixed in place. Following rectal and intracochlear temperature stabilization at 36.5–37.5°C, each animal was subjected to normothermia or hypothermia induction protocols. During the 2-h normothermia TTM protocol, all rectal and intracochlear temperatures in anesthetized animals varied within the target normothermic range of 36–38°C, averaging less than a 1°C change from baseline (Figure 1B). In contrast, during the hypothermia induction protocol, the neck collar provided efficacious and consistent mild hypothermia equally to both cochleae (~32–33°C), averaging a 4–6°C decrease from baseline (Figure 1C). Local hypothermia did not affect systemic temperature, which remained within 1°C of baseline measurements.

### 3.2 Post-noise hypothermia significantly reduced hearing threshold shifts

Female Brown Norway rats aged 15–20 weeks were divided into three groups (Figure 2A). First, all groups were noise-exposed under isoflurane anesthesia for 2 h at 105 dB SPL noise (4–8 kHz). Animals in the noise only group were allowed to recover in normothermic awake conditions, whereas animals assigned to normothermia and hypothermia treatment groups underwent a 2-h TTM protocols under ketamine/xylazine sedation. The administered ketamine doses throughout TTM were equal between groups (Student’s *t*-test, *p* = 0.2482). Obtained ABR thresholds were compared at all time points for all groups using a linear mixed model with fixed time, treatment and frequency effects, and random subject effect. Whole model factors accounted for 82% (=*R*^2^-adjusted) of the variance, including effects of stimulus frequency (*p* < 0.0001), treatment (*p* < 0.0001), time (<0.0001), and their two-way interactions (<0.0001). Tukey’s *post hoc* comparisons suggest statistically significant protection of hearing thresholds with post-noise Hypothermia TTM treatment when compared to normothermic TTM and noise only groups.

Figure 2A shows the experimental protocols and timelines for measurements in the three groups. The noise exposure resulted in significant combined temporary and permanent threshold shifts which were evident starting 1 DPN (Figure 2). Animals in the noise only group (n = 6) demonstrated significantly elevated threshold shifts at 1 DPN across the tonotopic map of normal hearing for Brown Norway rats. Normothermia animals had comparable threshold shifts post noise across the frequencies. In comparison, the hypothermia treated animals exhibited significant protection against noise-induced threshold shifts starting 1 DPN. *Post hoc* analysis with Bonferroni correction at 1 DPN threshold shifts showed significance for hypothermia treatment compared to noise only group at all frequencies (*p* < 0.01) and to normothermia treated group at click and low-to-mid frequencies (*p* < 0.01) as well as at 32 kHz (*p* < 0.05). Noise+Normothermia and noise only animals presented similar early temporary threshold shifts. At 28 DPN, animals without post-noise TTM averaged 35.8 ± 45.2 dB threshold shifts at 8 kHz, constituting a significant threshold elevation compared to TTM groups with observed 10.8 ± 4.4 and 5.0 ± 4.7 dB threshold shifts for respective post-noise normothermia and hypothermia. In summary, the one-time post-noise hypothermia treatment continued to preserve hearing thresholds up to the last day of testing (28 DPN). The hypothermia treated group was the only one that had recovery of hearing thresholds to their pre-noise baseline across the tonotopic map. Subsequent mixed-effect model analyses were performed for each experimental group to determine the progression of compound threshold shift from baseline (detailed in Table 1). Faster recovery toward baseline was observed with high-frequency hearing loss compared to low- and mid-frequencies, apart from hypothermia TTM animals that observed little-to-no changes at low-frequency regions. Animals receiving post-noise hypothermia TTM also showed quicker recovery to baseline within 7 DPN.

**Table 1:** Post-noise hypothermia TTM accelerates recovery of ABR thresholds to baseline.

Recovery		1 DPN			3 DPN			7 DPN			14 DPN			28 DPN		
	kHz	*M*	*SE*		*M*	*SE*		*M*	*SE*		*M*	*SE*		*M*	*SE*	
Noise – TTM	Ck	**37.5**	7.3	**	**24.0**	4.8	**	**18.3**	4.6	**	13.3	5.1		14.2	4.2	
	2	**35.8**	6.0	**	**16.0**	5.6	*	13.3	4.8		10.8	4.2		9.2	4.0	
	4	**38.3**	4.2	**	**20.0**	5.2	**	**15.8**	3.7	*	9.2	2.4		12.5	1.1	
	8	**46.7**	3.3	**	**33.0**	6.8	**	**35.8**	7.2	**	**30.8**	5.8	**	**35.8**	5.2	**
	16	**50.0**	1.8	**	**32.0**	6.8	**	**25.8**	5.1	**	**24.2**	4.0	**	**22.5**	7.2	**
	24	**45.8**	3.0	**	15.0	6.3		8.3	5.6		10.0	6.1		7.5	5.0	
	32	**55.8**	4.5	**	**32.0**	4.1	**	15.0	5.8		8.3	3.6		10.0	5.2	
Noise + Normo.	Ck	**26.7**	6.7	**	**24.2**	5.8	**	**20.8**	4.2	**	16.7	4.2		10.0	4.7	
	2	**20.8**	7.0	**	**17.5**	4.4	**	**18.3**	3.3	*	15.0	3.4		5.0	5.3	
	4	**38.3**	6.9	**	**28.3**	5.9	**	**21.7**	4.8	**	14.2	5.5		10.8	5.5	
	8	**48.3**	5.7	**	**35.0**	8.9	**	**28.3**	4.4	**	**23.3**	6.5	**	10.8	4.4	
	16	**33.3**	6.0	**	**21.7**	4.4	**	**20.0**	5.0	**	14.2	5.2		5.8	3.5	
	24	**35.8**	9.0	**	**21.7**	8.2	**	10.8	6.5		10.0	5.0		5.0	4.1	
	32	**43.3**	7.0	**	**26.7**	5.3	**	14.2	4.5		8.3	4.4		5.8	2.4	
Noise **+ Hypo.**	Ck	**11.0**	4.3	*	0.0	1.6		2.0	3.0		2.0	3.0		3.0	3.4	
	2	2.0	4.4		2.0	1.2		−2.0	3.4		−2.0	2.0		−2.0	2.0	
	4	3.0	2.5		1.0	1.0		2.0	2.0		0.0	1.6		1.0	2.4	
	8	**21.0**	4.0	**	**12.0**	3.7	**	8.0	3.4		7.0	4.1		5.0	4.7	
	16	**13.0**	5.1	**	3.0	2.5		0.0	3.5		3.0	2.5		3.8	1.3	
	24	**19.0**	5.8	**	2.0	2.5		2.0	3.0		0.0	1.6		−1.3	1.3	
	32	**23.0**	3.0	**	4.0	3.7		3.0	2.0		1.0	1.9		2.5	2.5	

Significance level.

** < 0.01, * < 0.05.

Summary of ABR threshold shifts from baseline obtained before and after exposure to 2 h of 105 dB noise. Results of mean ± SE ABR threshold shifts for noise only, normothermia, and hypothermia TTM animals are shown for all tested stimuli (click, 2–32 kHz). Group-specific statistical difference from pre-noise measurements was obtained at 1, 3, 7, 14, and 28 DPN (*p < 0.05, **p < 0.01). Significant values in bold. ABR Threshold Shifts up to 28 DPN. dB, decibels; Normo, *Normothermia*; Hypo, *Hypothermia*; DPN, days post-noise; Ck, Click; Mean ± SE.

### 3.3 Effects of noise and TTM on hair cell integrity

Cochleae were extracted for analysis of hair cell integrity after the final hearing evaluation at 28 DPN using FITC-Phalloidin staining No IHC loss was observed for all groups’ representative basal, middle, and apical regions (Figure 2C). The survival percentage of OHCs from all obtained microphotographs ranged from 83 to 100%. Between-group comparisons of OHC survival demonstrated no difference between the groups at the distinct cochlear sections (two-way ANOVA, treatment*cochlear section, *p* = 0.4982, angular transformation). *Unexposed control* animals showed the lowest total variance between samples with a grand mean of 99.1% survival in all regions. Grand averages for OHC survival in noise-exposed animals were 96.6 ± 1.2, 97.2 ± 0.9, and 96.0 ± 1.5% for hypothermic, normothermic, and noise only animals. The highest variability observed was in the basal region, where OHC survival was 96.1 ± 2.8, 95.6 ± 1.1, and 91.3 ± 3.4% for the same groups. No substantial OHC loss was indicated in any noise-exposed group compared to unexposed controls (*post hoc* Dunnett’s).

### 3.4 Hypothermia rescued noise-induced synaptopathy

**Figure 3:**
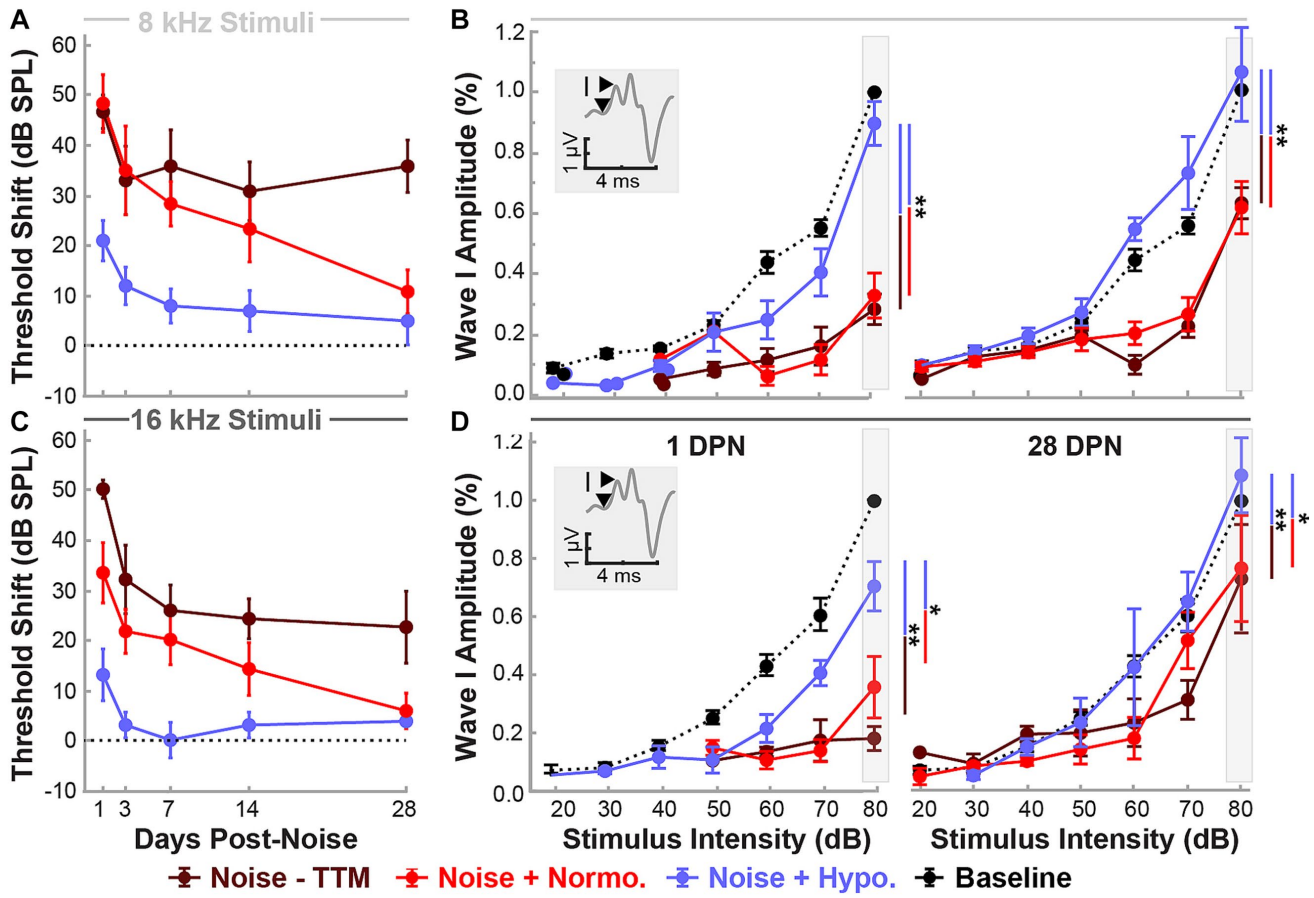
Hypothermic preservation of ABR Wave I amplitudes post-noise. ABR threshold elevations (mean ± SE) from respective baseline measurements are highlighted for two frequencies, 8 kHz (light gray, **A**) and 16 kHz (dark gray, **C**). Thresholds shifts from 1 to 28 DPN for noise (dark red), normothermia (red), and hypothermia treated animals (blue) are shown. The dotted line visualizes the baseline. Wave I amplitudes normalized to respective baseline measures at 80 dB are presented (mean ± SE) at stimulus intensity levels from 20 to 80 for 8 **(B)** and 16 **(D)** kHz pure-tone stimuli. Normalized baseline amplitude calculations (dotted line) are shown for comparison to normalized post-noise amplitudes at 1 and 28 DPN. Figure insets portray Wave I amplitude measurement from node to peak. Between-group comparisons at suprathreshold level of 80 dB are illustrated with vertical bars (*p < 0.05, **p < 0.01).

Changes in functional outcomes in our model were not a result of hair cell loss. To further investigate the neurophysiological pathology underlying these changes and the therapeutic benefits of preclinical intervention strategies for NIHL, we used ABR suprathreshold amplitude as a proxy measure of synaptopathy (Furman et al., 2013; Hashimoto et al., 2019; Aedo and Aguilar, 2020). ABR amplitudes at 8 and 16 kHz cochlear regions at 80 dB were analyzed to study the effects of treatment and stimulus intensity on ABR Wave I amplitudes with a mixed-effect model (Restricted Maximum Likelihood, REML). Figures 3A,C show the threshold shifts post-noise between the three groups of animals for 8 and 16 kHz stimuli over the 28 day period. Figures 3B,D show Wave I peak amplitudes at 1 and 28 DPN. At pre-noise baseline, the amplitudes between the three groups were similar (dotted line) and only revealed an effect of stimulus intensity as expected (*p* < 0.0001). A significant reduction in amplitudes was observed across stimulus intensities for the two noise groups. In comparison, the hypothermia treatment group revealed amplitudes that were similar to baseline. REML model fitting performed at a suprathreshold value of 80 dB revealed strong relations of treatment (*p* = 0.0003), time (*p* < 0.0001), and the interaction between time and frequency (*p* = 0.0354). At 28 DPN, a complete recovery of normalized suprathreshold Wave I amplitude was seen only in hypothermia treated animals, whereas noise only and noise+normothermia groups had significantly lower normalized amplitudes. Post-hoc comparisons for both 8 and 16 kHz stimuli indicated that Wave I was preserved with hypothermia TTM compared to both Noise-TTM and Noise+Normothermia TTM groups at both 1 and 28 DPN (*p* < 0.04).

### 3.5 Hypothermia treatment preserves synaptic puncta following noise exposure

**Figure 4:**
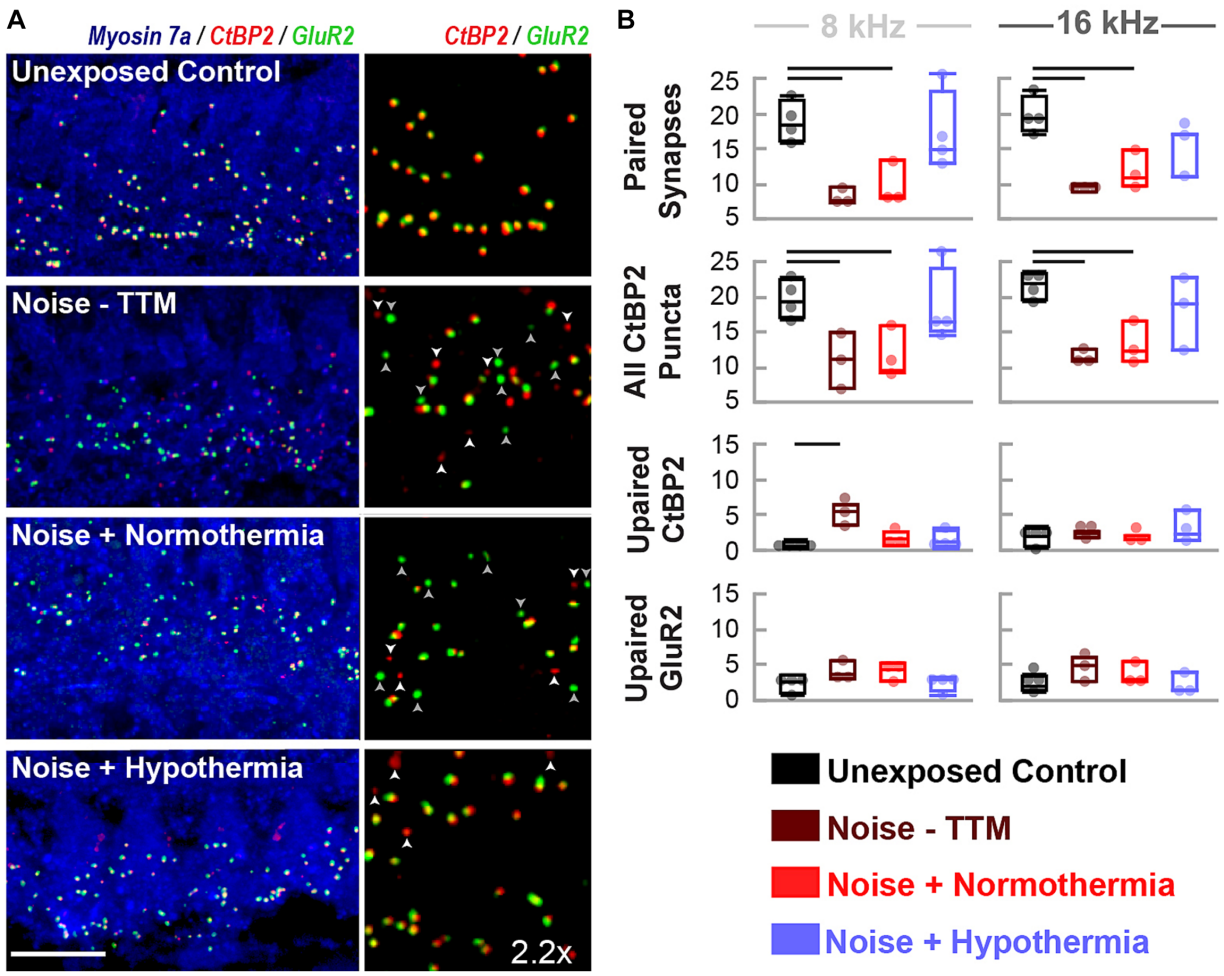
Hypothermia applied after synaptopathic noise exposure preserves pairing of pre-and post-synaptic puncta. **(A)** Confocal images of paired presynaptic ribbons (anti-CtBP2, red) and post-synaptic glutamate receptors (anti-GluR2, green) in the IHC area (anti-myosin VIIa, blue) were obtained for unexposed controls, noise, normothermia TTM, and hypothermia TTM animals at 24 h post-noise. Vertical panels depict the colocalization of CtBP2 and GluR2 labeled puncta with an enlarged (2.2x) view of the IHC area. Scale bar: 10 μm **(B)** Visually quantified, colocalized synapses and unpaired puncta per IHC were obtained for 8 and 16 kHz areas. Comparisons to control (black) animals were made for paired and unpaired puncta in noise-exposed animals without post-noise TTM (dark red) and with post-noise normothermia (red) and hypothermia (blue) (mean ± SE, *p < 0.05, **p < 0.01).

To measure synaptic puncta, histological measures of CtBP2 and GluR2 were quantified from cochlear sections corresponding to 8 and 16 kHz regions (Figure 4). Multi-group, multivariate analysis of variance (treatment*frequency) on paired and orphan puncta at the 8 and 16 kHz frequency regions suggested a robust treatment (*p* = 0.0003) and interaction (*p* = 0.01749) effect (Figure 4B). Paired synapse analyses revealed a treatment effect (*p* < 0.0001) but not for the frequency location (*p* = 0.6019, Interaction *p* = 0.7304). Tukey pairwise comparisons were made at each frequency location to illustrate between-group differences. Preservation of paired synapses with hypothermic intervention was pronounced at the 8 kHz region. The number of paired synapses for unexposed controls averaged 18.7 ± 1.4 synapses per IHC, whereas for hypothermia treated animals, they averaged 16.9 ± 2.9 synapses per IHC. In comparison, normothermic and noise only groups were significantly reduced to 9.7 ± 1.7 (*p* < 0.05) and 8.1 ± 0.6 (*p* < 0.05) synapses per IHC, respectively. For the 16 kHz region, the average synapse counts were 19.9 ± 1.3, 15.1 ± 1.9, 11.7 ± 1.6 (*p* < 0.05), and 9.45 ± 0.4 (*p* < 0.01) paired synapse counts per IHC for unexposed controls, hypothermia, normothermia, and noise only groups, respectively. Irrespective of pairing status, the CtBP2 puncta revealed that pre-synaptic ribbons reduced at both frequency locations the two noise groups (one-way ANOVA, *p* = 0.0031). CtBP2-labeled ribbons averaged 19.5 ± 1.8, 17.5 ± 1.8, 11.4 ± 2.1 and 10.8 ± 2.1 puncta per IHC for respective unexposed control, hypothermia, normothermia and noise-only animals at the 8 kHz region. For the 16 kHz region, the counts were 21.7 ± 1.8, 18.2 ± 2.1, 13.1 ± 2.1 and 11.4 ± 2.1 puncta per IHC, respectively. The number of orphan CtBP2 puncta also showed treatment (*p* = 0.0160) and treatment*frequency (*p* = 0.0175) differences (one-way ANOVA, *p* = 0.0104) with the results highlighting benefits of therapeutic hypothermia.

### 3.6 Long-term safety and efficacy of post-noise TTM

**Figure 5:**
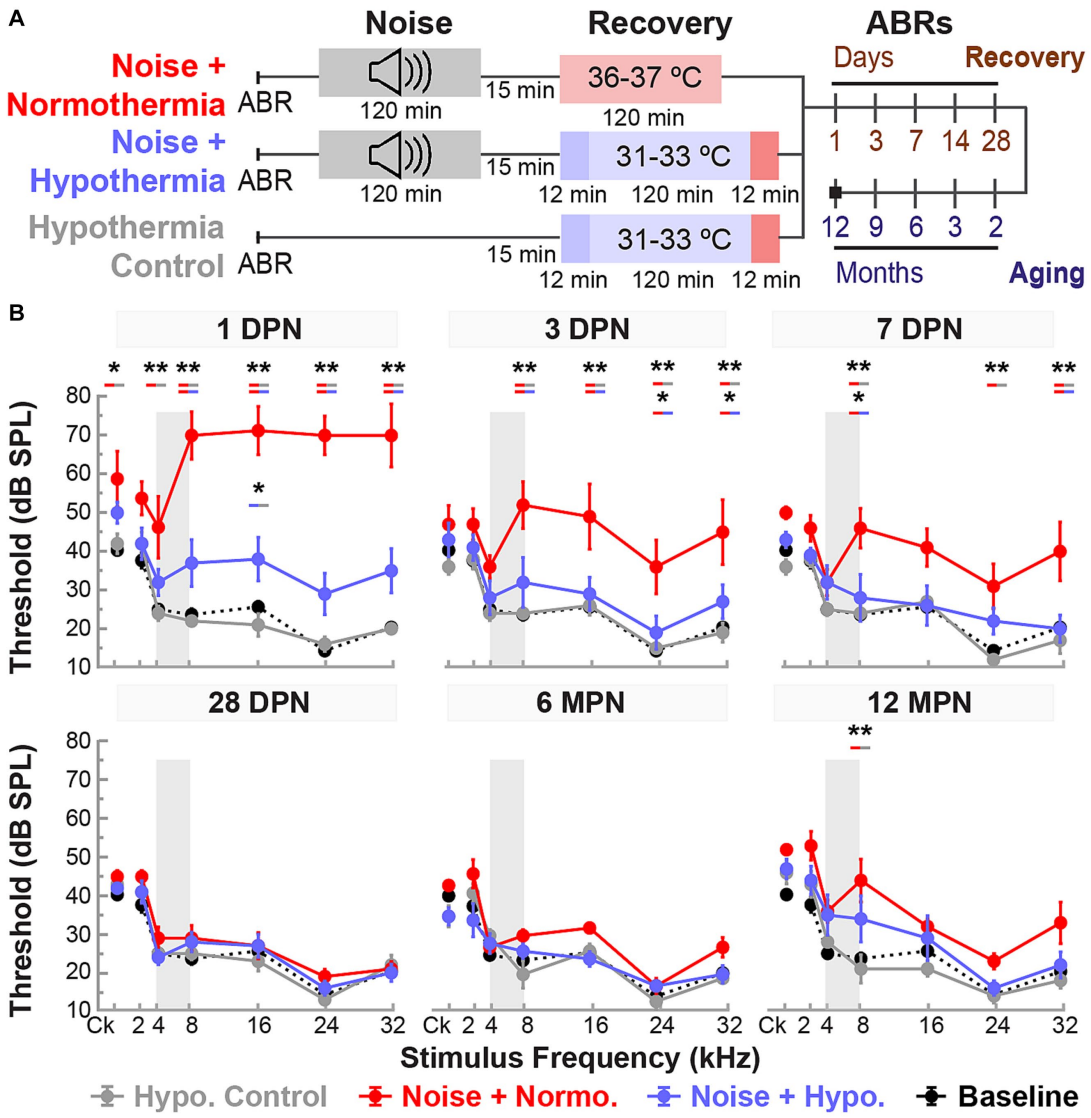
Post-noise hypothermic induction limits temporary hearing changes associated with accelerated age-related hearing loss. **(A)** Experimental protocol for the study of *Hypothermia TTM* long-term efficacy and safety. Animals in noise groups were subjected to 2-h continuous noise exposure (4–8 kHz, 105 dB) under isoflurane anesthesia before initiating *TTM* protocols (normothermia or hypothermia) under anesthesia. **(B)** Baseline (black dotted line) and post-procedure ABR thresholds (mean ± SE) are illustrated at multiple time points to observe the progression of hearing recovery between experimental groups. Early time points at 1, 3, 7, and 28 DPN were used to observe the acute recovery response, and later time points at 6 and 12 MPN to observe aging deterioration in previously noise-exposed animals. ABR thresholds were obtained for broadband (click, Ck) stimuli and pure tones ranging from 2 to 32 kHz. Gray bands depict the narrowband 4–8 kHz frequency composition of the 2-h noise exposure. Between-group comparisons are illustrated for each tested frequency and time point (REML, *p < 0.05, **p < 0.01).

We next performed long-term experiments which studied the effects of a one-time application of localized hypothermia on hearing thresholds and animal behavior over 12 months (Figure 5). In the long-term experiments, we also tracked hearing thresholds in a third, hypothermia only (no noise) group of rats (Figure 5A). Again, administered ketamine doses were comparable between the TTM groups (One-way ANOVA, *p* = 0.6789).

REML analysis of ABR thresholds included fixed effects for stimulus frequency (<0.0001), treatment (0.0005), and time (<0.0001), as well as their two-way interactions (<0.0001) and three-way interaction (*p* = 0.0060). As expected, the thresholds for hypothermia control group did not deviate from baseline, averaging threshold shifts within 5 dB of baseline measurements across all frequencies. The noise-exposed animals on the other hand exhibited pronounced temporary threshold shifts at 1 DPN, particularly at mid-to-high frequencies. For example, normothermia animals experienced threshold shifts between 45 to 56.3 dB for 8, 16, 24, and 32 kHz, respectively. In comparison, the hypothermia treatment group had threshold shifts between 9.3 to 16 dB only at the respective frequencies. *Post hoc* comparisons revealed elevated thresholds (*p* < 0.0001) at 1 DPN for noise+normothermia animals compared to both hypothermia treated and control animals (8, 16, 24, and 32 kHz).

Average threshold shifts from baseline at all tested frequencies and times post-noise are shown in Figure 5B and detailed in Table 2. Mixed-effect model analyses explained over 70% of the variance (R^2^, adj) for hypothermia control, normothermia, and hypothermia treatments. Significant fixed effects of time and frequency and their interaction effect were observed only in noise exposed animals. Highlighting the safety of therapeutic hypothermia, the MTH treated control animals exhibited no substantial changes in hearing sensitivity across the 12 months. Highlighting the efficacy of therapeutic hypothermia, a faster recovery was observed in hypothermia TTM animals. In this group, there was a relatively smaller threshold elevation across frequencies at 1 DPN. The thresholds recovered by 3 DPN and maintained across the 12 months. In contrast, temporary changes in hearing were observed up to 14 DPN in post-noise normothermia TTM animals. Thresholds of this group remained elevated at the culmination of the 12 MPN aging phase for 2, 8, and 32 kHz.

**Table 2:** ABR threshold shifts observed up to 12 months post-exposure to 105 dB Noise.

Recovery		1 DPN			3 DPN			7 DPN			14 DPN			28 DPN		
	Hz	*M*	*SE*		*M*	*SE*		*M*	*SE*		*M*	*SE*		*M*	*SE*	
Hypo. Control	Ck	1.0	2.4		−5.0	1.6		−5.0	1.6		−1.0	1.9		2.0	1.2	
	2 k	3.0	2.5		−1.0	1.9		−1.0	1.9		−1.0	2.9		2.0	2.5	
	4 k	2.0	3.0		2.0	2.5		3.0	3.4		6.0	2.9		3.0	3.0	
	8 k	2.0	2.0		4.0	1.9		3.0	2.5		7.0	3.4		5.0	1.6	
	16 k	−3.0	3.0		2.0	3.4		3.0	3.0		2.0	4.9		−1.0	1.9	
	24 k	4.0	1.0		3.0	1.2		0.0	1.6		4.0	1.9		1.0	1.9	
	32 k	−4.0	1.0		−5.0	1.6		−7.0	4.1		−1.0	2.4		−2.0	3.0	
**Noise +** Normo.	Ck	**20.0**	7.4	**	8.0	5.1		11.0	2.9		11.0	1.9		6.0	1.0	
	2 k	**17.5**	7.5	*	11.0	7.0		10.0	6.3		8.0	4.6		9.0	4.8	
	4 k	**18.8**	9.0	**	9.0	3.7		5.0	3.5		6.0	1.9		2.0	3.0	
	8 k	**45.0**	7.4	**	**27.0**	7.2	**	**21.0**	6.2	**	**18.0**	4.1	**	5.0	3.5	
	16 k	**46.3**	7.5	**	**25.0**	9.9	**	**17.0**	6.4	*	**16.0**	6.6	*	3.0	4.4	
	24 k	**56.3**	6.6	**	**20.0**	6.1	**	15.0	5.5		11.0	2.9		5.0	3.2	
	32 k	**53.8**	8.3	**	**27.0**	7.3	**	**22.0**	6.4	**	12.0	4.1		4.0	3.7	
Noise + Hypo.	Ck	**9.0**	3.3	**	2.0	4.6		2.0	4.1		−1.0	4.6		1.0	2.4	
	2 k	**4.0**	6.8	**	3.0	6.0		1.0	4.8		3.0	5.8		3.0	5.1	
	4 k	**6.0**	3.7	**	2.0	4.6		6.0	4.8		0.0	2.2		−2.0	2.5	
	8 k	**11.0**	7.0	**	6.0	6.6		2.0	6.0		6.0	5.8		2.0	3.4	
	16 k	**9.0**	5.6	**	0.0	4.5		−3.0	5.4		0.0	4.7		−2.0	3.4	
	24 k	**14.0**	6.2	**	4.0	5.8		7.0	4.6		5.0	3.5		1.0	2.9	
	32 k	**16.0**	7.0	**	8.0	6.6		1.0	3.7		4.0	4.0		1.0	2.4	

Aging		2 MPN			3 MPN			6 MPN			9 MPN			12 MPN		
	Hz	*M*	*SE*		*M*	*SE*		*M*	*SE*		*M*	*SE*		*M*	*SE*	
Hypo. Control	Ck	−4.0	2.9		−6.0	2.4		−6.0	2.9		−5.0	2.9		5.0	2.7	
	2 k	0.0	1.6		8.0	1.2		2.0	2.0		3.3	1.7		4.0	2.4	
	4 k	3.0	3.4		5.0	4.2		8.0	2.0		5.0	7.6		6.0	1.9	
	8 k	2.0	4.1		1.0	3.3		0.0	2.2		−3.3	1.7		1.0	2.9	
	16 k	3.0	5.6		4.0	2.9		2.0	3.7		3.3	6.0		−3.0	4.4	
	24 k	7.0	1.2		5.0	1.6		1.0	2.9		0.0	0.0		2.0	1.2	
	32 k	−5.0	2.7		−1.0	2.4		−5.0	0.0		−3.3	1.7		−6.0	2.9	
Noise + Normo.	Ck	3.0	3.4		−1.1	2.9		4.0	1.9		5.0	4.6		13.0	2.0	
	2 k	4.0	6.6		−1.0	5.3		10.0	2.2		8.8	5.5		**17.0**	3.4	*
	4 k	0.0	2.2		−1.0	1.0		0.0	0.0		5.0	2.0		9.0	3.7	
	8 k	6.0	5.1		5.0	4.2		5.0	2.2		5.0	4.6		**19.0**	6.0	**
	16 k	7.0	3.4		4.0	1.9		8.0	3.0		6.3	2.4		8.0	3.4	
	24 k	−3.0	3.4		2.0	3.4		1.0	3.7		−3.8	3.8		7.0	4.1	
	32 k	5.0	3.2		3.0	2.5		9.0	2.9		7.5	1.4		15.0	4.2	
Noise + Hypo.	Ck	−3.0	4.4		−7.0	6.0		−6.0	4.6		1.3	4.3		6.0	4.0	
	2 k	0.0	5.7		−2.0	5.6		−4.0	3.7		0.0	3.5		6.0	5.8	
	4 k	−1.0	2.9		3.0	4.1		2.0	2.5		−3.8	4.3		9.0	5.8	
	8 k	1.0	4.0		0.0	4.5		0.0	1.6		−6.3	6.9		8.0	5.8	
	16 k	−4.0	2.4		−5.0	1.6		−5.0	2.2		−6.3	1.3		0.0	5.9	
	24 k	3.0	3.4		1.0	2.9		2.0	2.0		−2.5	2.5		1.0	3.3	
	32 k	3.0	2.5		3.0	2.0		1.0	3.3		−1.3	2.4		3.0	3.4	

Significance level.

** < 0.01, * < 0.05.

Summary of ABR threshold shifts from baseline obtained before and after exposure to 2 h of 105 dB noise. Results of mean ± SE ABR threshold shifts for hypothermia control, noise+normothermia, and noise+hypothermia animals are shown for all tested stimuli (click, 2–32 kHz). In addition, group-specific statistical differences from baseline measurements were obtained at 1, 3, 7, 14, and 28 DPN within the acute recovery phase (tan) and at 2, 4, 6, 9, and 12 MPN for the early aging phase (blue). Differences from baseline are expressed for each frequency and time point (*p < 0.05, **p < 0.01). Significant values in bold. ABR Threshold Shifts up to 12 MPN. dB, decibels; Normo, *Normothermia*; Hypo, *Hypothermia*; DPN, days post-noise; Ck, Click; Mean ± SE.

### 3.7 One-time hypothermia application preserves Wave 1 amplitudes

**Figure 6:**
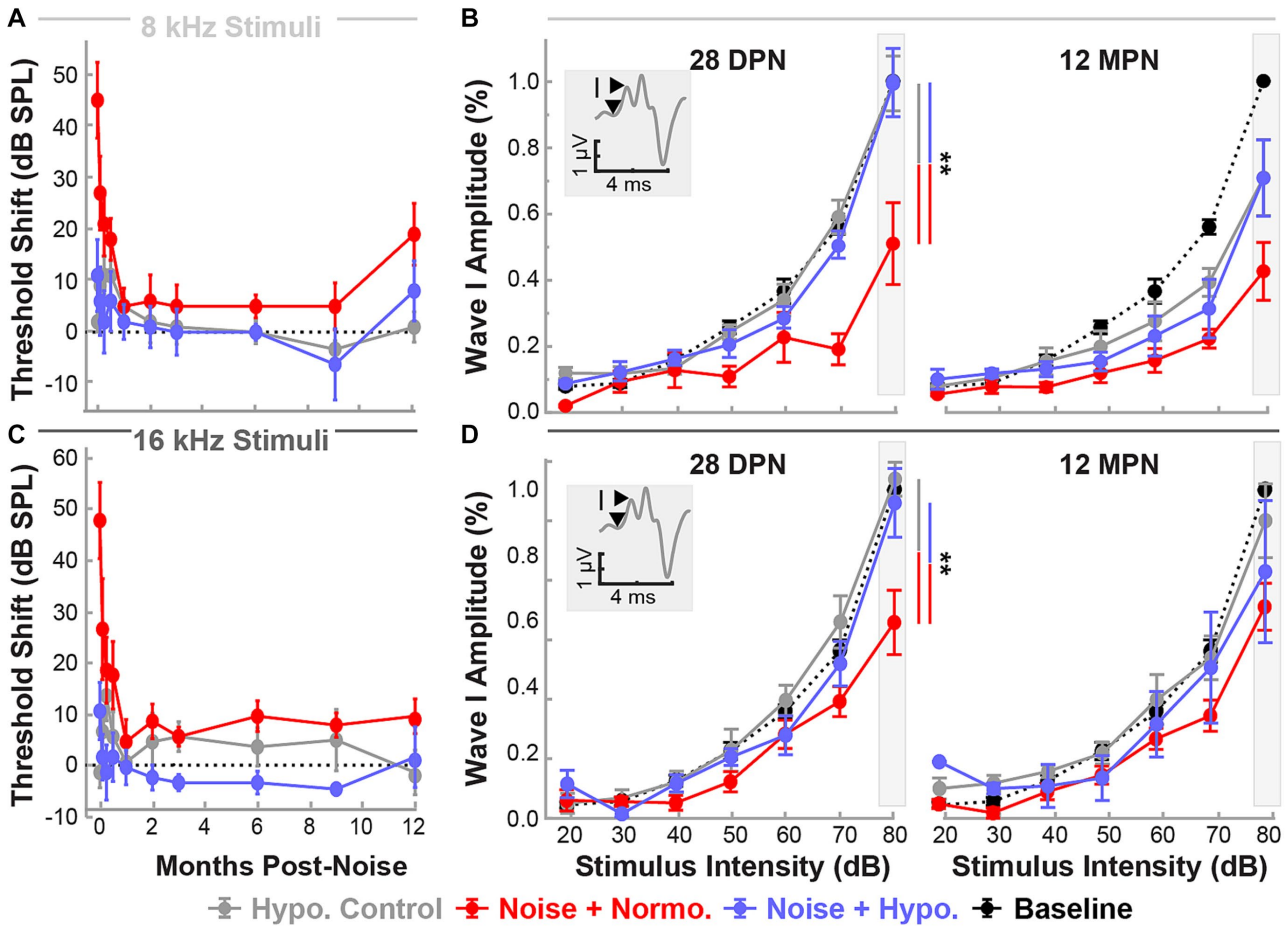
Post-noise localized hypothermic induction recovers suprathreshold ABR Wave I amplitude by 28 DPN. Recovery of ABR threshold up to 12 MPN is indicated by threshold shifts (mean ± SE), post-procedure thresholds with baseline threshold subtraction. Data are shown for frequencies 8 kHz (light gray, **A**) and 16 kHz (dark gray, **C**) for the three experimental groups hypothermia control (gray), noise+normothermia (red), and noise+hypothermia (blue). The dotted line depicts a difference from baseline threshold. Baseline-normalized Wave I amplitudes (mean ± SE) are depicted at stimulus intensities of 20 to 80 dB for respective middle frequencies (**B** – 8 kHz, **D** – 16 kHz). ABR amplitudes are normalized for each subject to its baseline amplitude at a maximal stimulus intensity of 80 dB SPL. Average Normalized baseline amplitudes for each frequency are exemplified with a black dotted line. Normalized post-procedure ABR amplitudes are compared at 28 DPN as the primary endpoint of acute noise recovery and 12 MPN as the final study endpoint. Figure insets illustrate Wave I peak and node demarcation for amplitude measurement. Between-group comparisons at suprathreshold level of 80 dB are illustrated with vertical bars (*p < 0.05, **p < 0.01).

Figure 6 shows normalized suprathreshold ABR Wave I amplitudes for 8 and 16 kHz stimuli for the end of the recovery (28 DPN) and aging phases (12 MPN). Results of REML analysis indicated fixed effects of treatment (*p* = 0.0022) and time (*p* = 0.0123). By 28 DPN, only normothermic animals had lasting wave amplitude reductions of 0.51 ± 0.12 and 0.61 ± 0.10 for 8 and 16 kHz stimuli. In comparison, hypothermia controls and hypothermia treated animals retained the wave 1 amplitudes near baseline (0.99 ± 0.08 and 0.99 ± 0.10 for 8 kHz and 1.04 ± 0.05 and 0.96 ± 0.10 for 16 kHz stimuli respectively). Post-hoc contrasts after the 28 DPN recovery phase indicated lower normothermic amplitudes compared to hypothermia Control (*p* = 0.0024) and noise-exposed (*p* = 0.0072) animals. However, by the end of the 12 MPN aging phase, wave I amplitudes at 8 kHz were 0.70 ± 0.12, 0.71 ± 0.12 and 0.43 ± 0.09 of their baseline values for hypothermia control, hypothermia treated, and normothermic groups. The normalized amplitudes for the same groups at 12 MPB for 16 kHz stimuli were 0.91 ± 0.11, 0.75 ± 0.21, and 0.65 ± 0.07. No differences between normalized amplitudes were observed after the 12 MPN aging phase (*post hoc* contrasts, *p* > 0.05). Comparing normalized wave IV amplitudes at the same time and frequencies revealed no between-group differences (REML, *p* > 0.05, Supplementary Figure S2).

### 3.8 Hypothermia treatment preserves spiral ganglion densities following noise exposure

**Figure 7:**
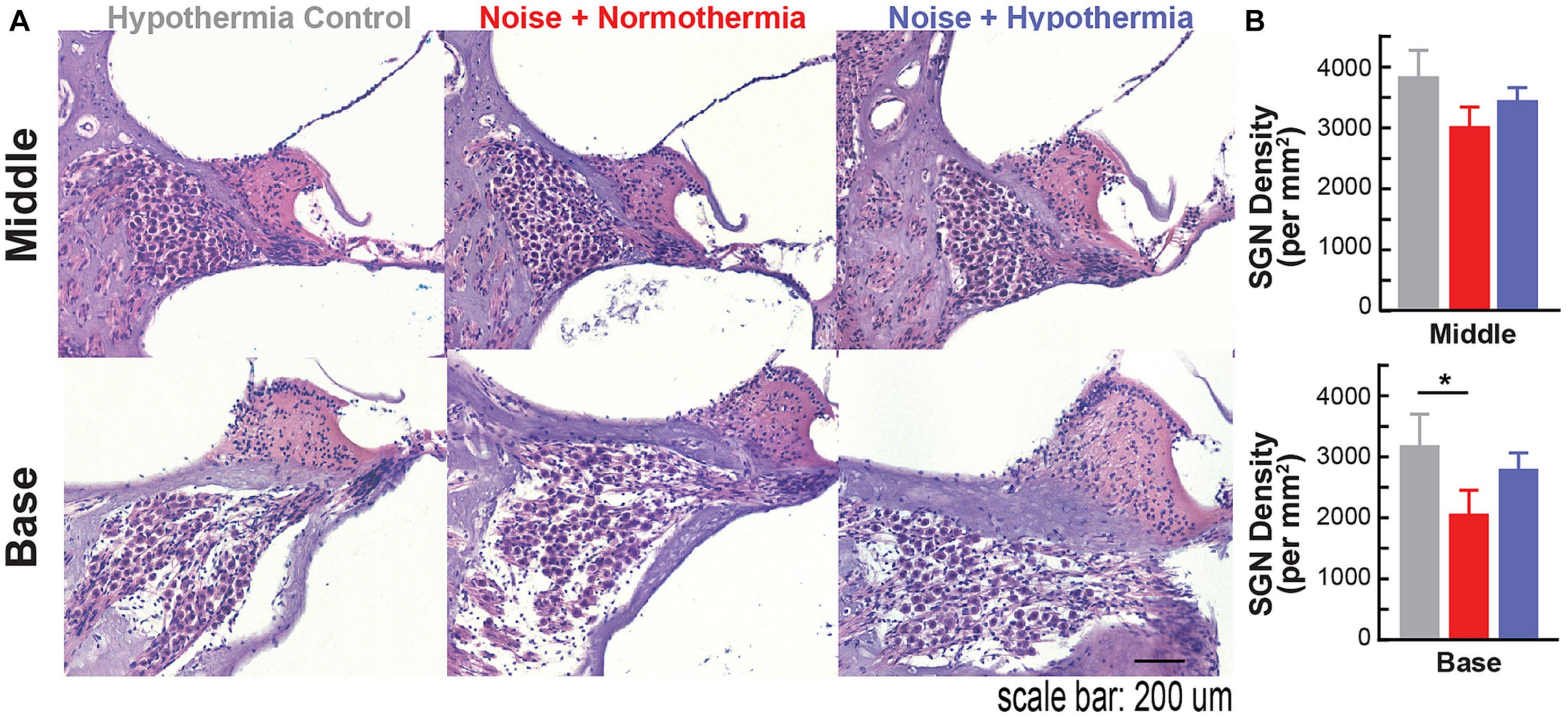
Spiral ganglion loss associated with acute early noise-exposure. **(A)** Hematoxylin/eosin (H&E) stained cross-section of the middle turn in Brown Norway rats. Image is from a representative 10 μm paraffin-embedded section imaged at 20X. H&E-stained cochlear cross-section across the modiolus display spiral ganglion neurons housed in Rosenthal’s canal at different cochlear turns. Scale bar: 100 μm. **(B)** Quantified number of SGNs in 10 μm mid-modiolar sections at the basal (top) and middle (bottom) turns were compared across hypothermia control (gray), normothermia (red), and hypothermia (blue) female brown Norway rats at 12 months post-noise (two-way ANOVA, *p < 0.05, **p < 0.01).

Following the experiment terminal point of 12 MPN, cochleae were extracted to assess spiral ganglion neuropathy (Figure 7). Spiral ganglion densities were calculated for non-exposed hypothermia control animals and noise-exposed normothermia and hypothermia TTM animals. SGNs were counted within Rosenthal’s canal for HE-stained cochlear sections (10 μm thickness). Figure 7A shows example sections from the middle and basal regions. Calculated SGN densities at the basal turn averaged 3,188 ± 514, 2,800 ± 265, and 2,064 ± 386 SGNs/mm^2^ for respective hypothermia control, noise+hypothermia, and noise+normothermia groups. For the same groups, densities at the middle turn were 3,839 ± 426, 3,450 ± 205, and 3,022 ± 313 SGNs/mm^2^. Comparison across the groups (two-way ANOVA, treatment*cochlear section, *p* = 0.0419) revealed a statistically significant decrease of neural density in noise+normothermia animals compared to hypothermia controls at the basal region (*post hoc* contrast, *p* = 0.0400).

## 4 Discussion

The present study demonstrates the efficacy of non-invasive, localized, and transient therapeutic hypothermia against NIHL in a preclinical model. Over the past decades, considerable progress has been made to characterize the cellular and molecular mechanisms underlying noise-induced sensorineural hearing loss pathophysiology. While early studies focused on hair cell and spiral ganglion loss and permanent hearing loss, more recent preclinical and clinical studies have focused on the peripheral neural substrate – synaptopathy and hidden hearing loss. Using well-established functional and histological techniques and a preclinical rodent model, we show that hypothermia can mitigate or delay the progression of early damaging effects of noise. In our study, ABR thresholds revealed significant rescue of functional hearing and hair cell function with hypothermia applied post-noise. At the same time, ABR Wave I amplitudes across treatment groups and histological analysis at 1 DPN show that MTH treatment reduced noise-induced cochlear synaptopathy that may not be detectable by pure-tone threshold analysis alone. Furthermore, we did not observe any negative side effects of the MTH treatment or placement of the cooling collar on the skin, hearing outcomes, or behavior of the rodents.

### 4.1 Protective effects of hypothermia

The target temperature of hypothermia application may play a significant role in the protective effects of hypothermia. For example, mild to moderate (35 to 30°C) temperatures are assumed to be protective, whereas profound (12 to 20°C) hypothermia can be toxic in cultured cortical neurons (Tymianski et al., 1998). In the present report, we limited the mild hypothermia target temperature to ~33°C and combined it with a longer duration and slow rewarming protocol to optimize its protective effects.

A previous study utilizing a mechanical cochlear implant trauma model observed that localized hypothermic induction protects against HC loss (Tamames et al., 2016). As a global modulator of multiple cellular and subcellular processes, temperature management may provide wide-ranging protection targeting apoptotic mechanisms that may result in HC death and glutamate excitotoxicity that can damage synaptic ribbon integrity (Sangaletti et al., 2022). Hair cell death following intense acoustic trauma may occur through several metabolic processes, with OHCs long considered to have increased vulnerability to acoustic overexposure. The current TTS/PTS-inducing noise exposure did not cause appreciable outer or inner hair cell loss at 28 DPN. ABR thresholds and suprathreshold amplitudes recovered over time across groups, but a more rapid and robust recovery was observed with the hypothermia treatment. Hypothermia treatment alone did not adversely impact function or structure, suggesting the safety of localized cooling.

### 4.2 Mechanisms for protective effects of hypothermia

**Figure 8:**
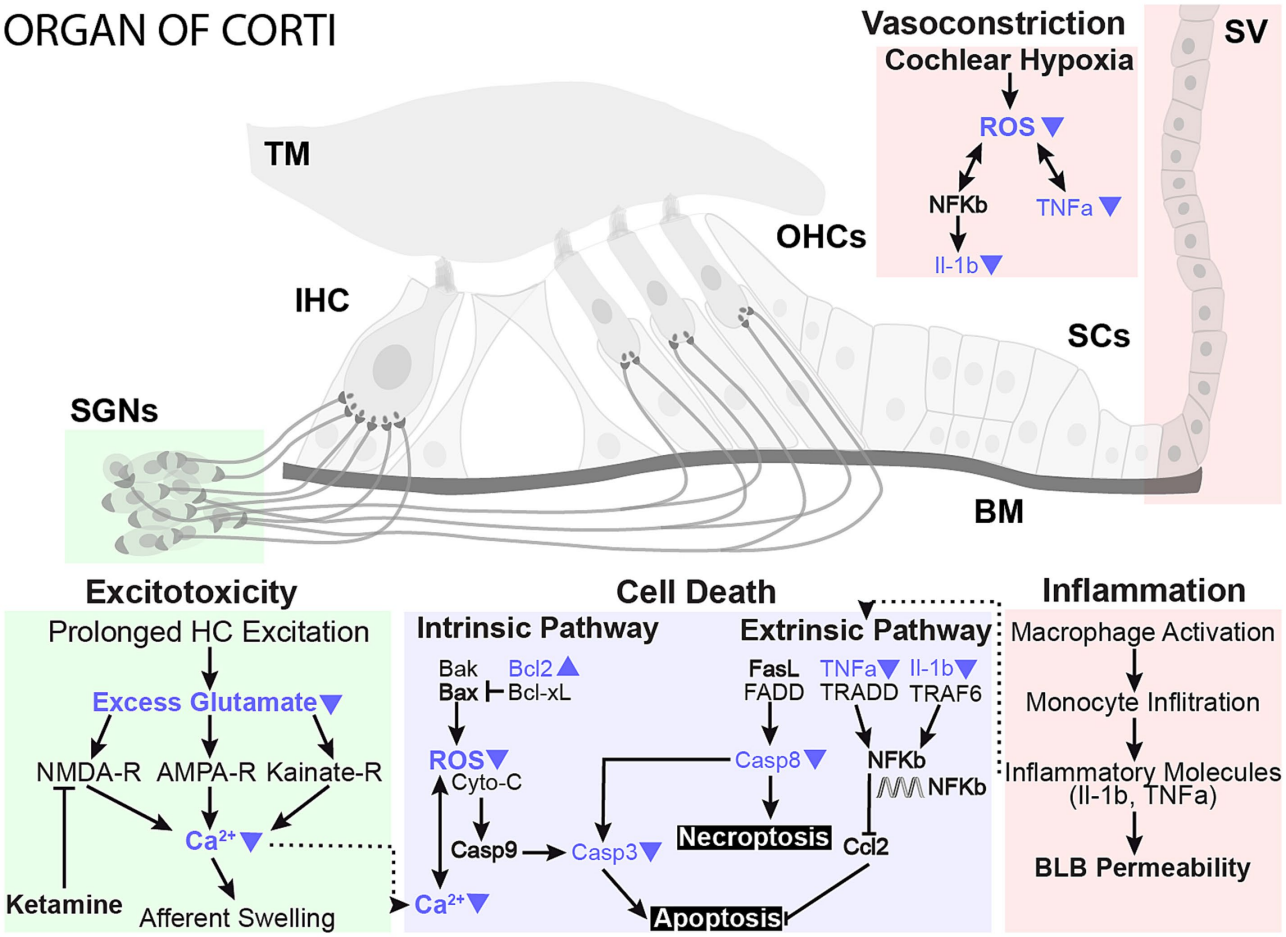
Proposed neuroprotective mechanisms of mild therapeutic hypothermia intervention in NIHL. The working model highlights prominent damage pathways involved in NIHL and potential sites of MTH action that likely play a protective role in preserving sensory hair cells and synaptic elements. Studies have shown that NIHL can activate apoptosis and necroptosis cell death pathways in hair cells and lead to glutamate excitotoxicity, negatively impacting synaptic elements. Conversely, MTH has been shown to have broad neuroprotective effects against ischemic and excitotoxic damage. The protective effects of MTH treatment on several NIHL damage pathways have been highlighted with blue arrows.

A wide range of cellular processes could contribute to the protective benefits of localized hypothermia on NIHL (Figure 8). One mechanism for noise-induced damage involves impaired mitochondrial function and increased oxidative stress (Henderson et al., 2006). Exposure to loud sounds can cause intense metabolic activity in cells, which increases free radical formation, including reactive oxygen species (ROS) from the mitochondrion (Henderson et al., 2006). Noise exposure-induced changes in calcium homeostasis can lead to ROS release into the cytoplasm, which can subsequently lead to irreversible cochlear damage and loss of sensory cells through necrotic and apoptotic activities that continue days after noise exposure (Baker and Staecker, 2012; Furness, 2015). ROS also contributes to modulation of pro-inflammatory cytokines such as interleukins (Fujioka et al., 2006; Wakabayashi et al., 2010; Arslan et al., 2017) and tumor necrosis factor (TNF-a, Van Wijk et al., 2006; Keithley et al., 2008). A recent study described a tight temperature dependence of mitochondrial calcium buffering that is associated with MTH neuroprotection in hypoxic–ischemic injury (Sosunov et al., 2022). In a companion paper, ATP-synthase genes and expression of other genes of the mitochondrial respiratory chain complex are upregulated following hypothermia treatment, further implicating mitochondrial function in a therapeutic role (Sabatino Rincon et al., 2023, in review).

MTH may also directly promote the reduction of inflammatory cytokines. One study showed that MTH significantly down-regulated pro-inflammatory interleukins, such as TNF-α, and beneficially up-regulated BCL-2 in a cortical microelectrode implant model (Dugan et al., 2020). Bcl-2 promotes cell survival and inhibits apoptosis by inhibiting free radical production, preventing mitochondrial cytochrome c release, and blocking caspase activation. RNA-seq further demonstrated that cytokines are among the most downregulated genes in the hypothermia-treated group, suggesting that modulation of inflammation may be a primary mechanism for hypothermic protection (Sabatino Rincon et al., 2023, in review, Figure 8).

### 4.3 Noise-induced synaptopathy and the protective role of hypothermia

Preclinical mammalian studies have revealed significant cochlear synaptopathy – a disappearance of synaptic connections between inner hair cells and auditory nerve fibers – occurs with aging and after acute noise exposure, despite a recovery of auditory sensitivity following a temporary shift (Kujawa and Liberman, 2009; Lin et al., 2011; Jensen et al., 2015; Valero et al., 2017). Cochlear synaptopathy is largely accompanied by a permanent reduction in the supra-threshold amplitude of the ABR wave I, analogous to decreased spiral ganglion activity within the cochlear nerve (Kujawa and Liberman, 2009). While there is a gradually progressing loss of synapses in the cochleae with aging, noise-induced trauma increases the rate of this loss (Kujawa and Liberman, 2006; Fernandez et al., 2015). However, standard hearing evaluation tools, ABR thresholds or shifts (or audiograms in clinic), may fail to detect synaptopathy and the consequent hidden hearing loss (Aedo and Aguilar, 2020; Henry, 2021). In the current proof-of-concept study, we used a noise protocol (2 h, 105 dB) that produced a combination of temporary and permanent threshold shifts (TTS, PTS). In addition to the ABR thresholds, we analyzed potential synaptopathy at early time points following TTS-inducing noise exposure using ABR Wave I amplitude measurements and with a detailed immunohistochemical assessment of pre- and post-synaptic densities at two cochlear frequency regions where the acute threshold shift was maximal (8 and 16 kHz). After an acute recovery phase, a lasting suprathreshold reduction of ABR Wave I amplitudes was observed within the noise and normothermic animals. The animals receiving hypothermia primarily recovered to their baseline within 28 DPN. In addition, we observed a significant rescue of the paired synaptic elements with post-noise hypothermia when compared across treatment groups at 1 DPN.

Prior studies, primarily conducted in mice, highlight that following TTS-inducing noise exposure, there is an acute swelling of cochlear neuron dendrites and cell bodies. (Wang et al., 2002). As evidenced by the recovery of ABR thresholds in our study and previous reports, the swelling thought to contribute to these temporary changes in hearing sensitivity likely subsides by 28 DPN (for review, see Kujawa and Liberman, 2015; Ryan et al., 2016). However, the possibility of delayed degeneration of the spiral ganglion neurons that follows transient hearing loss associated with synaptic ribbon loss and disorganization (Kujawa and Liberman, 2009) is still to be determined. To test the efficacy of a one-time application of hypothermia in mitigating both the acute synaptopathy and long-term neuronal degeneration, we extended our experiments to include effects of aging – up to 12 months post-noise. Our results, matching those published previously in mice and rats, revealed a decreased neural density in cochleae with animals receiving noise exposure compared to controls. This neuropathy was primarily observed at the basal cochlear region. While the direct comparison between noise-exposed animals receiving post-noise hypothermia and normothermia was not established in terms of synaptic elements, early measures of paired pre- and post-synaptic terminals and total presynaptic puncta were only reduced in animals not receiving post-trauma cooling treatment.

While the mechanisms underlying noise-induced synaptopathy are still not fully understood, glutamate agonism or intense noise exposure can result in excitotoxic swelling of afferent endings (Pujol et al., 1985; Puel et al., 1998). Synaptic genes with diverse functions have altered expression in noise+normothermia and noise+hypothermia conditions (Sabatino Rincon et al., 2023, in review). Swelling of inner hair cell auditory synapses (type I synapses) is associated with both temporary and permanent auditory threshold shifts and is closely followed by neural degeneration (Jensen et al., 2015; Kujawa and Liberman, 2015). Ischemia, noise exposure, and aminoglycoside treatment all increase glutamate concentration in the cochlear periphery, which may result in excitotoxicity that could aggravate hearing loss and drive synaptic disruption (Hakuba et al., 1997; Forge and Water, 1999; Duan et al., 2000; Matsuda et al., 2000). Prior study focusing on the post-ischemic application of mild hypothermia reported attenuated hearing loss and neuropathy that was associated with hypothermic reduction of glutamate efflux at IHC synapses (Hyodo et al., 2001). Overall, our results support a protective role of one-time mild therapeutic hypothermia post-noise.

### 4.4 Sex-dependent effects

Prior clinical and animal studies of hearing loss have reported sex-specific variations in hearing preservation and injury response (see review Villavisanis et al., 2020). In this current study, no significant differences between ABR baseline thresholds or Wave I and IV amplitudes were observed between males and females. Age-matched male animals were included to observe sex differences in noise-induced injury and the long-term therapeutic benefit of hypothermic intervention. Interestingly, the current study found no sex-specific effects on noise-induced threshold shifts within each experimental group, despite generally reduced changes in hearing sensitivity after acute noise exposure observed in females compared to age-matched males (see Supplementary Figures S1–S5 and Supplementary Table S1).

ABR threshold differences found between the noise-exposed experimental groups included treatment effects that were present with REML analysis with sex as a factor that remained in subsequent sex-specific analyses. Our ABR amplitude results, however, suggest that there may be synaptopathic hypothermic protection afforded only to females with a comparison of suprathreshold ABR wave I amplitudes. Further studies detailing hair cell loss and synapse densities compared across male and female animals within early recovery time points must be evaluated for further discussion. Various physiological differences may also be attributed to sex and hormonal variations during the estrous cycle and aging, including changes in cochlear vasculature (Reimann et al., 2011) and sympathetic activity (Vicente-Torres and Gil-Loyzaga, 2002). Altogether, the results of these studies emphasize the need to tailor paradigms of hypothermic treatment, including dosing and timing of induction and rewarming rate, in a sex-dependent manner. Further work establishing sex differences in injury pathways may help tailor specific guidelines for the hypothermic intervention of noise-induced hearing loss.

### 4.5 Role of anesthetics in noise exposure and recovery

This study utilized anesthetized animals to maintain animal positions for stable and targeted noise injury and treatment. Anesthetic agents, including isoflurane and ketamine used in this study, can influence peripheral and central auditory physiology, including ABR thresholds (Santarelli et al., 2003; Cederholm et al., 2012; Ruebhausen et al., 2012). Several studies have established the otoprotective effects of sedation during noise exposure (Kim et al., 2005; Chung et al., 2007). Though isofluorane or ketamine have not been explicitly tied to any distinct pathway for hearing preservation, the roles of NMDA antagonism (Giraudet et al., 2002; Yang et al., 2011), peripheral vasodilation (Kum et al., 2018; Ceylan et al., 2019), and stapedial muscle relaxation (Simmons, 1960) have all been proposed as viable otoprotective strategies. Given the well-characterized reduction of cochlear blood flow with intense noise (Shin et al., 2019), the coupling of anesthetic-induced peripheral vasodilation and glutamatergic excitotoxicity protection may produce synergistic protection against noise-induced injury in the organ of Corti.

The noise only group allowed us to evaluate the neuroprotection of ketamine anesthesia without intracochlear hypothermia. Ketamine alone did not protect from acute loss of paired synapses and presynaptic ribbons observed with immunohistological assessment at 1 DPN. However, there was a general recovery of supra-threshold ABR Wave I amplitude compared to non-treated animals. These results show that hypothermia treatment, and not a secondary effect from anesthesia, is globally driving the recovery of hearing sensitivity.

### 4.6 Limitations

Potential hypothermic vasoconstriction and rewarming-induced complications are associated with hypothermia (Koehn et al., 2020). Moderate to severe hypothermia can affect metabolism and oxygen consumption, the availability of oxygen, and blood parameters like partial pressure of gases, electrolytes, and pH (Polderman, 2009). However, the frequency and severity of these complications is largely influenced by the depth (moderate-to-severe hypothermia), the duration, or the extent of cooling (systemic induction). Consequent studies may be beneficial to characterize the level-dependent vasoactivity of localized cochlear hypothermia. The current localized approach with a mild therapeutic target temperature of 33–34°C was utilized to decrease these associated risks. Our protocol strictly controlled the rate of rewarming, informed by our experimental results and extensive literature on hypothermia (Alva et al., 2004, 2013), but may be further optimized by extension of rewarming phase.

## 5 Conclusion

Our results suggest that post-noise hypothermic rescue of hearing thresholds and ribbon synapses may provide long-term benefits to hearing health. Further work to maximize neurotherapeutic benefit may depend on understanding the mechanisms of neuroprotection, indicating methods of protocol optimization, or suggesting potential neuroprotective agents for adjunctive therapy. Nevertheless, this non-invasive, non-pharmaceutical application of hypothermia and targeted temperature management will be clinically valuable as a therapeutic against hearing loss caused by noise-induced inner ear insult.

## Data availability statement

The raw data supporting the conclusions of this article will be made available by the authors, without undue reservation.

## Ethics statement

The animal study was approved by the University of Miami Institutional Animal Care and Use Committee. The study was conducted in accordance with the local legislation and institutional requirements.

## Author contributions

SRi: Conceptualization, Data curation, Formal analysis, Funding acquisition, Investigation, Methodology, Software, Visualization, Writing – original draft, Writing – review & editing. AR: Data curation, Writing – review & editing. RS: Data curation, Methodology, Writing – review & editing. WD: Writing – review & editing. MH: Writing – review & editing. CK: Methodology, Resources, Writing – review & editing. SRa: Conceptualization, Data curation, Formal analysis, Funding acquisition, Investigation, Methodology, Project administration, Resources, Software, Supervision, Validation, Visualization, Writing – original draft, Writing – review & editing.

## Footnotes

^1^ https://www.masseyeandear.org/research/otolaryngology/eaton-peabody-laboratories/histology-core
