# Supplementary material for: Targeted therapeutic hypothermia protects against noise induced hearing loss
Source: Front Neurosci. 2024 Jan 16;17:1296458. doi: 10.3389/fnins.2023.1296458 (PMC10826421; doi:10.3389/fnins.2023.1296458)
Supplement: Supplementary file 1 [file Table_1.DOCX]

Supplementary Material

# Supplementary Data

**Supplementary Figure 1. Biological Sex Comparison of Baseline ABR Thresholds and Amplitudes.** (A) ABR thresholds in juvenile (15-20 weeks-old) Brown Norway rats were compared between males (open marker) and females (filled marker). ABR testing stimuli include broadband click (Ck) and pure tones at low (2, 4 kHz), middle (8,16 kHz), and high (24, 32 kHz) frequencies. (B) Comparison of male and female ABR node-to-peak amplitudes for Wave I are shown for middle frequencies, 8 and 16 kHz, at incremental stimuli intensity from 20 to 80 dB. Figure insets highlight node and peak demarcation for Wave I.

**Supplementary Figure 2. Post-Noise Hypothermia TTM limits temporary threshold elevation in male animals.**ABR thresholds (mean±SE) are depicted for all evaluated time points up to 28 days post-procedure. Average baseline thresholds for all male animals are indicated by a black dotted line to aid in the visualization of approximated full recovery (REML, *p < 0.05, **p<0.01).

**Supplementary Figure 3. Limited effect of TTM on post-procedure suprathreshold ABR Wave I amplitudes in male animals.**(A) Progression of threshold shift from baseline (mean±SE) for 8 (light grey, A) and 16 kHz (dark grey, C) pure-tone stimuli are illustrated at all evaluated post-procedure time points up to 12 MPN. Thresholds shifts are indicated for experimental groups. The dotted line indicates the normalized baseline. (B, D) Wave I amplitudes (mean±SE) for respective groups at 28 DPN and 12 MPN are portrayed at stimulus intensity levels from 20-80 dB for 8 kHz (B) and 16 kHz (D) pure tone stimuli. Average baseline amplitudes across all groups are indicated with a dotted line. Figure insets illustrate Wave I node-to-peak amplitude measurement. Between-group comparisons at suprathreshold level of 80 dB are illustrated with vertical bars (*p < 0.05, **p<0.01).

**Supplementary Figure 4. Spiral Ganglion Densities at 12 MPN in Male Brown Norway Rats.**Calculated SGN densities (mean±SE) obtained from H&E staining of Rosenthal’s Canal at basal (left) and middle (right) turns. SGN densities are observed for experimental groups in male Brown Norway rats at 12 months post-exposure to TTS-inducing noise.

| **Supplementary Table 1. Threshold Shifts observed up to 12 MPN in Male animals** | | | | | | | | | | | | | | | | | |
| --- | --- | --- | --- | --- | --- | --- | --- | --- | --- | --- | --- | --- | --- | --- | --- | --- | --- |
| **Recovery** | | | **1 DPN** | |  | **3 DPN** | |  | **7 DPN** | |  | **14 DPN** | |  | **28 DPN** | |  |
|  | Hz |  | *M* | *SE* |  | *M* | *SE* |  | *M* | *SE* |  | *M* | *SE* |  | *M* | *SE* |  |
| **Hypo. Control** | Ck |  | -2.0 | 3.4 |  | -1.0 | 4.8 |  | 3.0 | 4.6 |  | 2.0 | 5.1 |  | 0.0 | 5.5 |  |
|  | 2 |  | -1.0 | 4.3 |  | -2.0 | 2.0 |  | 6.0 | 3.3 |  | 2.0 | 4.4 |  | 4.0 | 2.9 |  |
|  | 4 |  | -1.0 | 2.9 |  | 1.0 | 4.8 |  | 5.0 | 2.7 |  | 4.0 | 3.3 |  | 4.0 | 2.9 |  |
|  | 8 |  | -3.0 | 4.1 |  | 1.0 | 5.8 |  | -1.0 | 2.9 |  | 4.0 | 3.7 |  | 2.0 | 3.0 |  |
|  | 16 |  | -1.0 | 2.4 |  | -4.0 | 2.4 |  | 3.0 | 1.2 |  | 1.0 | 3.7 |  | 0.0 | 3.5 |  |
|  | 24 |  | 3.0 | 2.5 |  | -2.0 | 3.4 |  | 0.0 | 3.2 |  | -1.0 | 4.0 |  | -2.0 | 1.2 |  |
|  | 32 |  | 6.0 | 1.0 |  | 2.0 | 2.0 |  | -3.0 | 2.0 |  | -2.0 | 1.2 |  | 1.0 | 1.9 |  |
| **Noise + Normo.** | Ck |  | **24.0** | 2.9 | ** | **24.0** | 3.7 | ** | **21.0** | 4.0 | ** | **17.0** | 3.7 | ** | 7.0 | 4.6 |  |
|  | 2 |  | **23.0** | 3.4 | ** | **18.0** | 3.0 | ** | **13.0** | 2.5 | * | **14.0** | 2.9 | * | 5.0 | 3.5 |  |
|  | 4 |  | **24.0** | 4.8 | ** | **17.0** | 1.2 | ** | 12.0 | 2.5 |  | 10.0 | 1.6 |  | 5.0 | 2.2 |  |
|  | 8 |  | **49.0** | 4.8 | ** | **35.0** | 3.5 | ** | **26.0** | 5.6 | ** | **23.0** | 3.7 | ** | 8.0 | 2.5 |  |
|  | 16 |  | **45.0** | 5.5 | ** | **21.0** | 4.8 | ** | **22.0** | 3.4 | ** | 12.0 | 2.5 |  | 4.0 | 2.9 |  |
|  | 24 |  | **56.0** | 4.0 | ** | **22.0** | 2.5 | ** | **13.0** | 2.0 | * | **15.0** | 3.5 | ** | 5.0 | 1.6 |  |
|  | 32 |  | **56.0** | 2.4 | ** | **27.0** | 4.1 | ** | **23.0** | 1.2 | ** | **16.0** | 1.9 | ** | 3.0 | 1.2 |  |
| **Noise + Hypo.** | Ck |  | **14.0** | 1.9 | ** | 3.0 | 4.1 |  | 4.0 | 4.3 |  | 7.0 | 2.5 |  | 1.0 | 2.9 |  |
|  | 2 |  | 6.0 | 1.9 |  | 6.0 | 1.9 |  | 3.0 | 2.5 |  | 3.0 | 2.5 |  | 4.0 | 2.4 |  |
|  | 4 |  | **12.0** | 2.5 | * | 3.0 | 1.2 |  | 7.0 | 3.0 |  | 6.0 | 2.4 |  | 3.0 | 1.2 |  |
|  | 8 |  | 8.0 | 4.9 |  | 3.0 | 3.4 |  | 8.0 | 2.0 |  | 6.0 | 3.3 |  | 2.0 | 2.0 |  |
|  | 16 |  | **17.0** | 1.2 | ** | 11.0 | 3.3 |  | 7.0 | 3.0 |  | 8.0 | 4.1 |  | 2.0 | 4.9 |  |
|  | 24 |  | **20.0** | 3.5 | ** | **14.0** | 2.9 | ** | 8.0 | 2.5 |  | 7.0 | 3.4 |  | 7.0 | 3.0 |  |
|  | 32 |  | **23.0** | 4.9 | ** | **16.0** | 1.0 | ** | 7.0 | 3.7 |  | 8.0 | 2.5 |  | 0.0 | 2.7 |  |
|  |  |  |  |  |  |  |  |  |  |  |  |  |  |  |  |  |  |
| **Aging** | | | **2 MPN** | |  | **3 MPN** | |  | **6 MPN** | |  | **9 MPN** | |  | **12 MPN** | |  |
|  | Hz |  | *M* | *SE* |  | *M* | *SE* |  | *M* | *SE* |  | *M* | *SE* |  | *M* | *SE* |  |
| **Hypo. Control** | Ck |  | 2.0 | 6.2 |  | -4.0 | 3.7 |  | 1.0 | 4.8 |  | 1.0 | 5.3 |  | 1.3 | 5.5 |  |
|  | 2 |  | 7.0 | 3.7 |  | 4.0 | 1.9 |  | 5.0 | 5.0 |  | 6.0 | 3.7 |  | 7.5 | 5.2 |  |
|  | 4 |  | 8.0 | 1.2 |  | 2.0 | 2.5 |  | 5.0 | 4.2 |  | 4.0 | 4.8 |  | 7.5 | 4.8 |  |
|  | 8 |  | 5.0 | 3.5 |  | 3.0 | 2.0 |  | 4.0 | 3.3 |  | 5.0 | 3.5 |  | 10.0 | 7.4 |  |
|  | 16 |  | 0.0 | 4.5 |  | 4.0 | 3.3 |  | 2.0 | 1.2 |  | 1.0 | 3.7 |  | 3.8 | 3.1 |  |
|  | 24 |  | 1.0 | 2.9 |  | 4.0 | 4.0 |  | -2.0 | 2.5 |  | 1.0 | 4.8 |  | 2.5 | 3.2 |  |
|  | 32 |  | -1.0 | 3.3 |  | 4.0 | 1.9 |  | 5.0 | 2.7 |  | 7.0 | 2.0 |  | 5.0 | 2.0 |  |
| **Noise + Normo.** | Ck |  | 8.0 | 5.4 |  | 9.0 | 3.7 |  | 13.8 | 6.3 |  | 12.5 | 5.2 |  | **21.3** | 3.8 | ** |
|  | 2 |  | 2.0 | 2.0 |  | 4.0 | 1.9 |  | 11.3 | 4.3 |  | 11.3 | 5.9 |  | **16.3** | 2.4 | ** |
|  | 4 |  | 3.0 | 2.0 |  | 6.0 | 1.9 |  | 11.3 | 3.1 |  | **13.8** | 3.1 | * | 11.3 | 3.1 |  |
|  | 8 |  | 10.0 | 3.2 |  | 10.0 | 4.7 |  | 11.3 | 4.3 |  | **17.5** | 6.6 | ** | **21.3** | 5.5 | ** |
|  | 16 |  | 1.0 | 1.9 |  | 5.0 | 1.6 |  | 8.8 | 3.1 |  | 3.8 | 3.8 |  | 11.3 | 2.4 |  |
|  | 24 |  | 5.0 | 1.6 |  | 8.0 | 2.0 |  | 1.3 | 2.4 |  | 5.0 | 0.0 |  | **15.0** | 4.1 | ** |
|  | 32 |  | 1.0 | 2.4 |  | 3.0 | 2.5 |  | 8.8 | 1.3 |  | 7.5 | 4.3 |  | **21.3** | 5.5 | ** |
| **Noise + Hypo.** | Ck |  | 6.0 | 2.9 |  | -1.0 | 1.0 |  | 2.5 | 3.2 |  | 3.8 | 3.8 |  | 11.3 | 4.3 |  |
|  | 2 |  | 1.0 | 1.0 |  | 1.0 | 1.9 |  | 1.3 | 2.4 |  | 5.0 | 2.9 |  | 12.5 | 3.2 |  |
|  | 4 |  | 2.0 | 1.2 |  | 4.0 | 1.0 |  | 3.8 | 3.1 |  | 2.5 | 1.4 |  | **15.0** | 2.0 | ** |
|  | 8 |  | -3.0 | 1.2 |  | -1.0 | 2.4 |  | 2.5 | 4.3 |  | -3.8 | 3.1 |  | 7.5 | 6.0 |  |
|  | 16 |  | 3.0 | 3.7 |  | 1.0 | 4.0 |  | 2.5 | 6.0 |  | 7.5 | 4.3 |  | 3.8 | 7.2 |  |
|  | 24 |  | 2.0 | 4.4 |  | 5.0 | 1.6 |  | 5.0 | 2.0 |  | 6.3 | 2.4 |  | 7.5 | 4.3 |  |
|  | 32 |  | 1.0 | 2.9 |  | 3.0 | 1.2 |  | 2.5 | 4.3 |  | 1.3 | 1.3 |  | 3.8 | 3.8 |  |
| Significance Level | | | | |  |  |  |  |  |  |  |  |  |  |  |  |  |
| **<.01, *<0.05 | | | |  |  |  |  |  |  |  |  |  |  |  |  |  |  |

**Supplementary Table 1.**Summary of ABR threshold shifts from respective baseline measurements at multiple post-procedure time points for male animals. ABR thresholds with baseline subtraction are reported as threshold shifts (mean±SE) for Hypothermia Control, Noise+Normothermia, and Noise+Hypothermia animals. Threshold shifts are shown for click (Ck) and pure-tone stimuli (2-32 kHz) at all measured time points, including the acute recovery phase (1-28 DPN, tan) and the early aging phase (2-12 MPN, blue). Group-specific post-procedure thresholds were compared to their respective baseline measurements indicating significant changes from pre-Noise and pre-TTM values (REML, *p < 0.05, **p<0.01).

ABR Threshold Shifts up to 12 MPN

dB: decibels, Normo: *Normothermia*, Hypo: *Hypothermia*, DPN: days post-noise, Ck: Click, Mean±SE.
