# Supplementary figures and images for: Targeted therapeutic hypothermia protects against noise induced hearing loss
Source: Front Neurosci. 2024 Jan 16;17:1296458. doi: 10.3389/fnins.2023.1296458 (PMC10826421; doi:10.3389/fnins.2023.1296458)

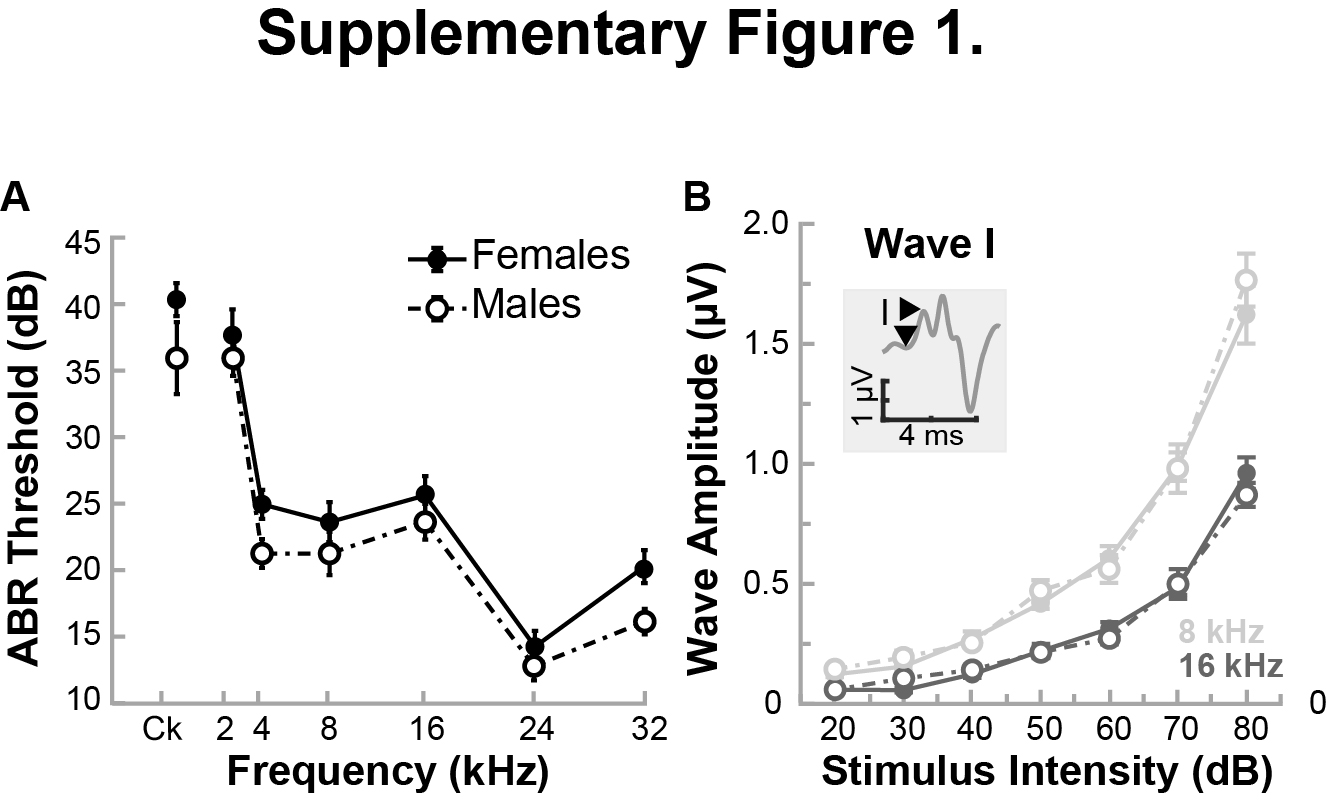

Supplement: Supplementary file 2 [file Figure_9.JPEG]

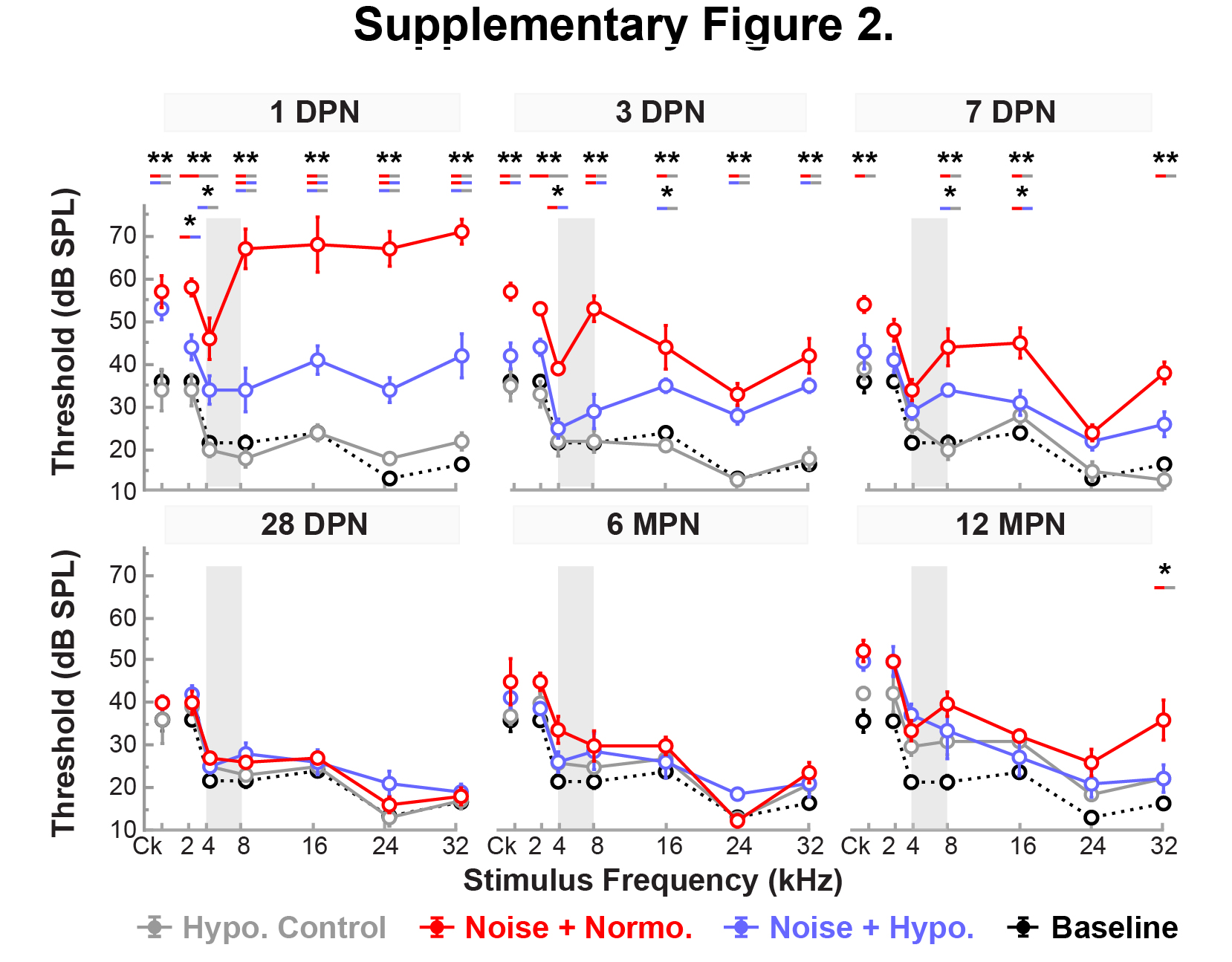

Supplement: Supplementary file 3 [file Figure_10.JPEG]

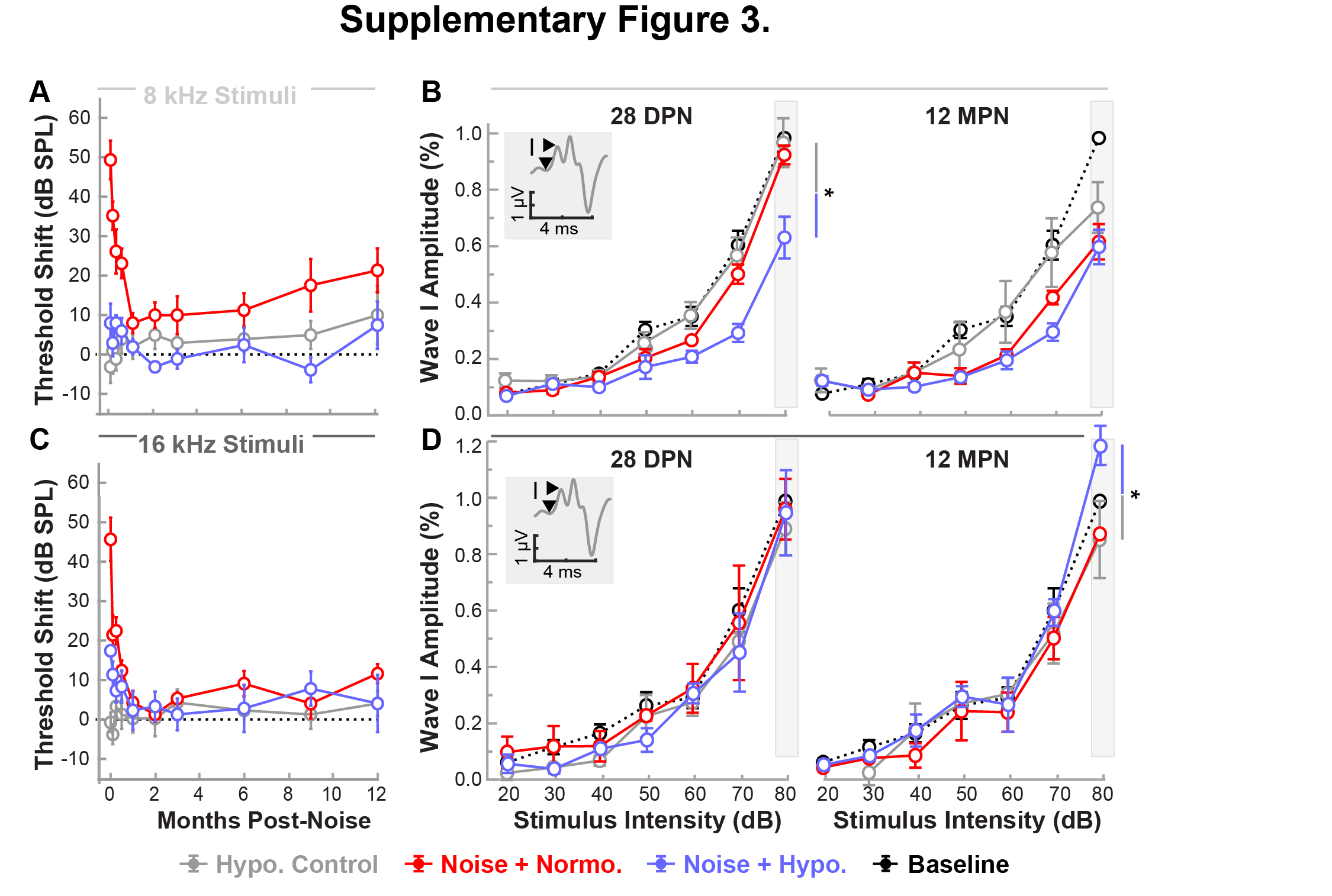

Supplement: Supplementary file 4 [file Figure_11.JPEG]

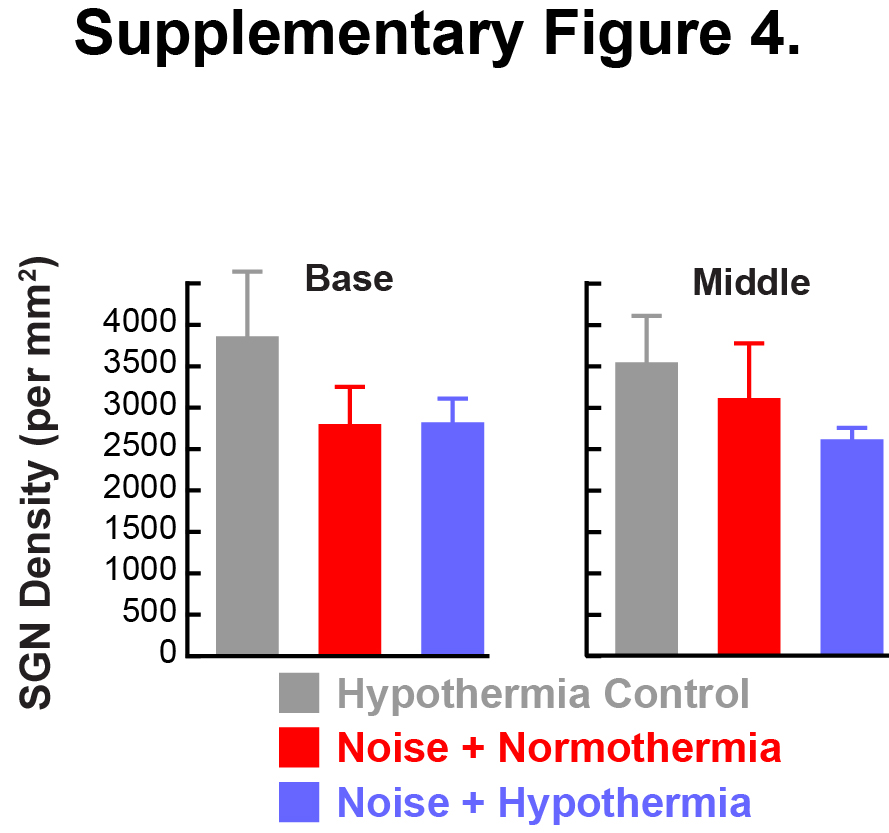

Supplement: Supplementary file 5 [file Figure_12.JPEG]
